# An empirical Bayes approach to normalization and differential abundance testing for microbiome data

**DOI:** 10.1186/s12859-020-03552-z

**Published:** 2020-06-03

**Authors:** Tiantian Liu, Hongyu Zhao, Tao Wang

**Affiliations:** 1 Department of Bioinformatics and Biostatistics, Shanghai Jiao Tong University, 800 Dongchuan Road, Shanghai, 200240 China; 2 Department of Biostatistics, Yale University, 300 George Street, New Haven, 06511 USA; 3 SJTU-Yale Joint Center for Biostatistics and Data Science, Shanghai Jiao Tong University, 800 Dongchuan Road, Shanghai, 200240 China; 4 MoE Key Lab of Artificial Intelligence, Shanghai Jiao Tong University, 800 Dongchuan Road, Shanghai, 200240 China

**Keywords:** Bayesian shrinkage, Differentially abundant OTUs, MetagenomeSeq, Phylogeny-aware analysis, Rarefying

## Abstract

**Background:**

Advances in DNA sequencing have offered researchers an unprecedented opportunity to better study the variety of species living in and on the human body. However, the analysis of microbiome data is complicated by several challenges. First, the sequencing depth may vary by orders of magnitude across samples. Second, species are rare and the data often contain many zeros. Third, the specimen is a fraction of the microbial ecosystem, and so the data are compositional carrying only relative information. Other characteristics of microbiome data include pronounced over-dispersion in taxon abundances, and the existence of a phylogenetic tree that relates all bacterial species. To address some of these challenges, microbiome analysis workflows often normalize the read counts prior to downstream analysis. However, there are limitations in the current literature on the normalization of microbiome data.

**Results:**

Under the multinomial distribution for the read counts and a prior for the unknown proportions, we propose an empirical Bayes approach to microbiome data normalization. Using a tree-based extension of the Dirichlet prior, we further extend our method by incorporating the phylogenetic tree into the normalization process. We study the impact of normalization on differential abundance analysis. In the presence of tree structure, we propose a phylogeny-aware detection procedure.

**Conclusions:**

Extensive simulations and gut microbiome data applications are conducted to demonstrate the superior performance of our empirical Bayes method over other normalization methods, and over commonly-used methods for differential abundance testing. Original R scripts are available at GitHub (https://github.com/liudoubletian/eBay).

## Background

It is well known that microbes interact with their human host. The human microbiome, which refers to the collection of microbes and their genetic information in the human body, contributes to healthy human physiology and development, and dysbiosis of microbial communities is linked to many diseases, such as obesity, type 2 diabetes, and inflammatory bowel disease [1–3]. Host genetics and environmental factors, in turn, affect the health and diversity of the human microbiome [4, 5]. However, the mechanisms underlying human health and disease remain largely unknown because of the complexity and dynamics of microbial communities. In order to understand the taxonomic composition and biological function of microbiomes, high-throughout sequencing technologies and advanced bioinformatics tools are now routinely employed in microbiome studies [6]. For example, marker gene analysis involves extracting DNA from primary samples, sequencing a highly variable region, and clustering sequence reads into Operational Taxonomic Units (OTUs) by sequence similarity (e.g., 97%). The evolutionary relationships among OTUs can also be inferred, by using a reference database, or by inferring the phylogenetic tree de novo [7].

Like differential expression analysis in microarray studies, one fundamental task in microbiome studies is differential abundance analysis, that is, to detect OTUs or species that have differential abundance between two or more experimental conditions, e.g., health versus disease [8]. Although differential expression analysis has been extensively studied, methods designed for continuous microarray data are not directly applicable for discrete microbiome data. The problem is further complicated by inherent characteristics of microbial community sequencing data [9]. In particular, the total reads per sample, known as the sequence depth or library size, can vary by orders of magnitude, and some OTUs are rare and therefore the data matrix is sparse. Consequently, there is a need to develop specialized analytical tools for microbiome data. Microbiome analysis workflows often begin with some type of normalization. Two commonly-used normalization approaches are rarefying, which subsamples the data without replacement to uniform sequence depth across samples, and total sum scaling, which divides read counts by the total count in each sample and bases downstream analyses on relative abundances [10]. While these two methods work well for the purpose of ordination, they often result in a high rate of false positives when testing for differentially abundant species [11]. Although rarefying is a recommended option in major data analysis toolkits [12, 13], it is inadmissible because it throws away some data and ignores the compositionality [10]. Microbiome data are compositional because the abundance of an OTU in a specimen is not the abundance of the corresponding taxon in the microbial ecosystem [14]. The special feature of compositional data is that a composition carries only relative abundance information.

Total sum scaling conditions on sequence depth and results in compositional data, i.e., raw proportions that sum up to 1. Since the data points map to a simplex rather than the Euclidean space, standard data analysis techniques, such as the t-test, are invalid. Instead of using the proportions directly, methods for analyzing compositional data all involve some type of transformation, the most common of which is the log-ratio transformation [15, 16]. Once the unit-sum constraint is removed, classical statistical methods apply, with care and proper interpretation to transformed data. Indeed, log-ratio-based inferences are increasingly popular in downstream microbiome analyses [14, 17–19].

Note that the raw proportions from total sum scaling are operationally equivalent in every way to the original count data when log-ratio transformed. One major problem with this naive scaling normalization technique [20] is when the normalized data have zeros, the log transformation is problematic. One approach to this issue is to replace the zero by a small positive value and re-normalize the data. Nevertheless, the choice of the constant is problem-dependent and its effect on the results is not well-studied [21]. Zero replacement is an active area of research, and statistically rigorous methods have emerged in the literature. For example, [22] and [23] respectively developed a non-parametric approach and a parametric treatment for imputing zeros. More recently, motivated by the fact that raw proportions from total sum scaling are maximum likelihood estimates of the unknown parameters under the multinomial model, [24] and [25] proposed replacement techniques from a Bayesian point of view. Assuming a Dirichlet prior for the set of proportions, a zero value is replaced by its posterior Bayesian estimate. The Bayesian method gives an estimate of the true composition, and hence can be viewed as a model-based alternative to total sum scaling.

The posterior Bayesian estimator shrinks the maximum likelihood estimator towards the mean vector of a Dirichlet prior. The smoothed estimates are more accurate than the raw proportions for OTUs with extremely high or low read counts. However, the obvious drawback of the existing methods is that a uniform prior is used, and therefore the shrinking point is uninformative. In addition, the prior is applied to single data points, but the observations may have a lot in common, and these similarities can be used to learn from the experience of others [26]. In this paper, we propose an empirical Bayes approach to normalization. Rather than adopting an uninformative prior, we assume that the parameters of the Dirichlet distribution is unknown, and we estimate them by using all observations in the data set. In addition to uneven sequence depth, data sparsity, and compositionality, the proposed method is designed to address over-dispersion and phylogeny.

It is known that microbiome data, and sequencing data in general, are over-dispersed, and that the multinomial distribution does not allow for over-dispersion. Over-dispersion is also a natural consequence of the data laying on the simplex. To account for the excess variation, the Dirichlet-multinomial (DM) distribution is commonly used in practice [27, 28]. DM is an analytically tractable compound distribution. This is a consequence of the fact that the Dirichlet distribution is a conjugate distribution to the multinomial distribution. The DM parameters are the hyper-parameters in the Dirichlet prior. We estimate these parameters from OTU counts by maximum likelihood. Then, we plug-in the estimates into the prior distribution, and normalize the data using the posterior mean. We further extend our method by incorporating phylogeny into the analysis. This is accomplished by using a tree-based extension of DM, called the Dirichlet-tree multinomial (DTM) distribution [29, 30]. Loosely speaking, DTM is a product of independent local DMs on internal nodes of the phylogenetic tree. While DM intrinsically imposes a negative correlation structure among bacterial counts, DTM allows for both positive and negative correlations [31].

## Results

We generated bacterial counts from a DM or DTM model, with the true vector of proportions ***π*** estimated based on a real dataset [32], which contains the counts of 60 taxa from 1897 samples, together with a phylogenetic tree describing the evolutionary relationship among these taxa. We note that, as mentioned earlier, in microbial ecology studies compositionality is not something imposed by post sequencing processing, and so microbiome data are compositions off the machine.

### Simulations without tree information

We first generated taxa abundance data from the DM model, with an over-dispersion parameter *θ*=0.15. We set the sample size *n*_1_=*n*_2_=50 and the number of taxa *p*=40. The sequencing depth was drawn uniformly from 5000 to 50000. Denote ***ϕ***_*k*_=(*ϕ*_*k*1_,...,*ϕ*_*kp*_)^T^ as the vector of true proportions in group *k*∈{1,2}. Initially, we generated ***ϕ***_1_=***ϕ***_2_ as a random sample from the 60-dimensional vector ***π***. We then normalized it to have unit sum, and varied the relative abundances of 6 taxa as follows:

1 $$ \left\{ \begin{array}{lr} \pi_{ks} \leftarrow \pi_{ks}+0.05\beta, & \\ \pi_{kt}\leftarrow \pi_{kt}-0.05\beta, &\\ \end{array} \right.  $$

where *s*∈{1,22,23}, *t*∈{7,11,40} for *k*=1, *s*∈{7,11,40}, *t*∈{1,22,23} for *k*=2, and *β*∈{0.01,0.15,0.2,0.25,0.3,0.35} represents the degree of difference between two groups. We further explored the impact of over-dispersion. We fixed *β*=0.25 and varied *θ*∈{0.05,0.1,0.15,0.2,0.25,0.3}.

**Fig. 1:**
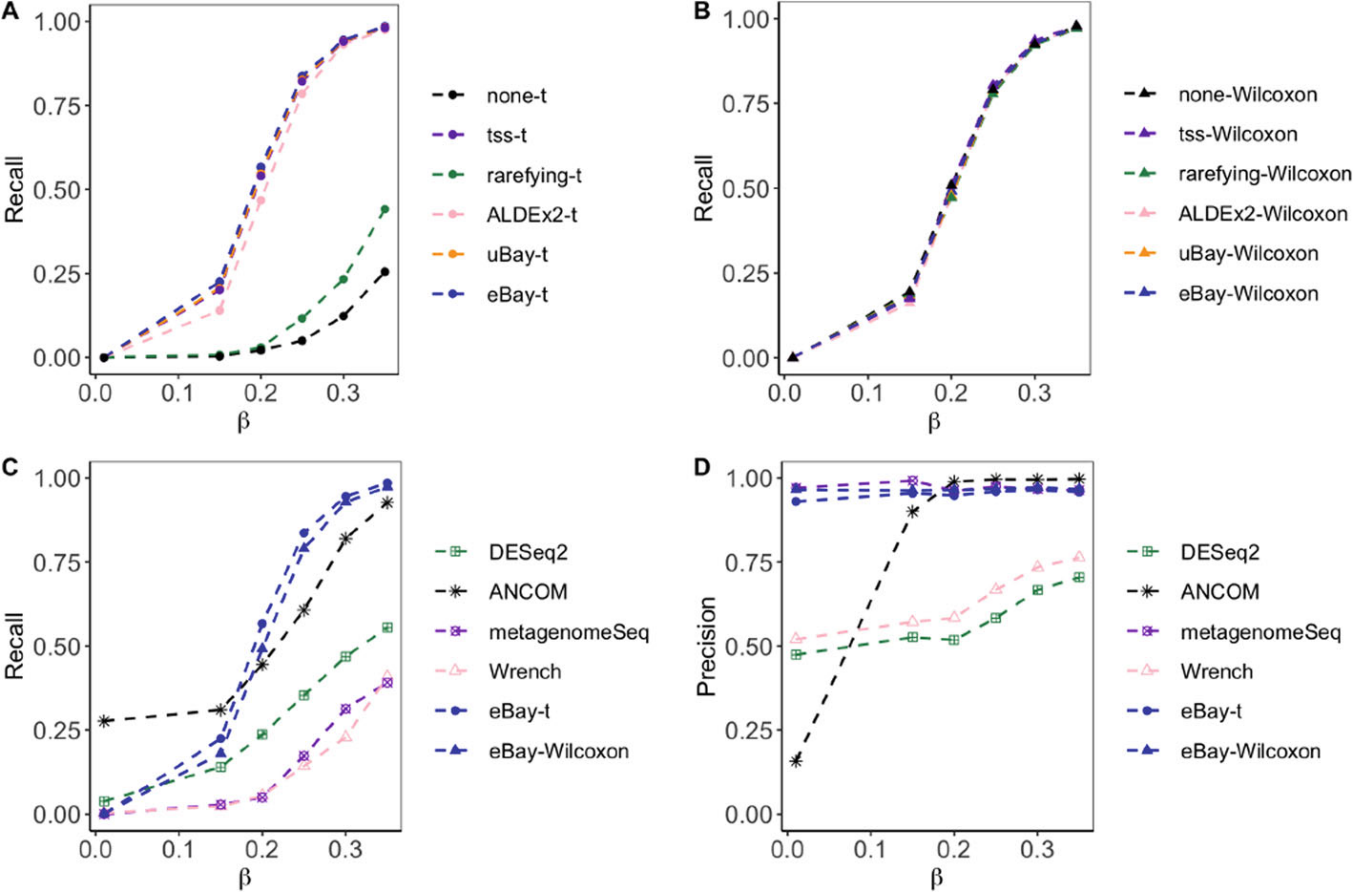
Comparison of recall and precision with data from DM across different *β*. To detect differentially abundant taxa, we simulated 100 data sets from the DM model with *θ*=0.15 and *β*∈{0.01,0.15,0.2,0.25,0.3,0.35}. **a** and **b** Recall of t-test and Wilcoxon rank sum test with various normalization methods. **c** and **d** Recall and precision of DESeq2, ANCOM, metagenomeSeq, Wrench, and those of t-test and Wilcoxon rank sum test, both applied after counts were normalized by eBay

**Fig. 2:**
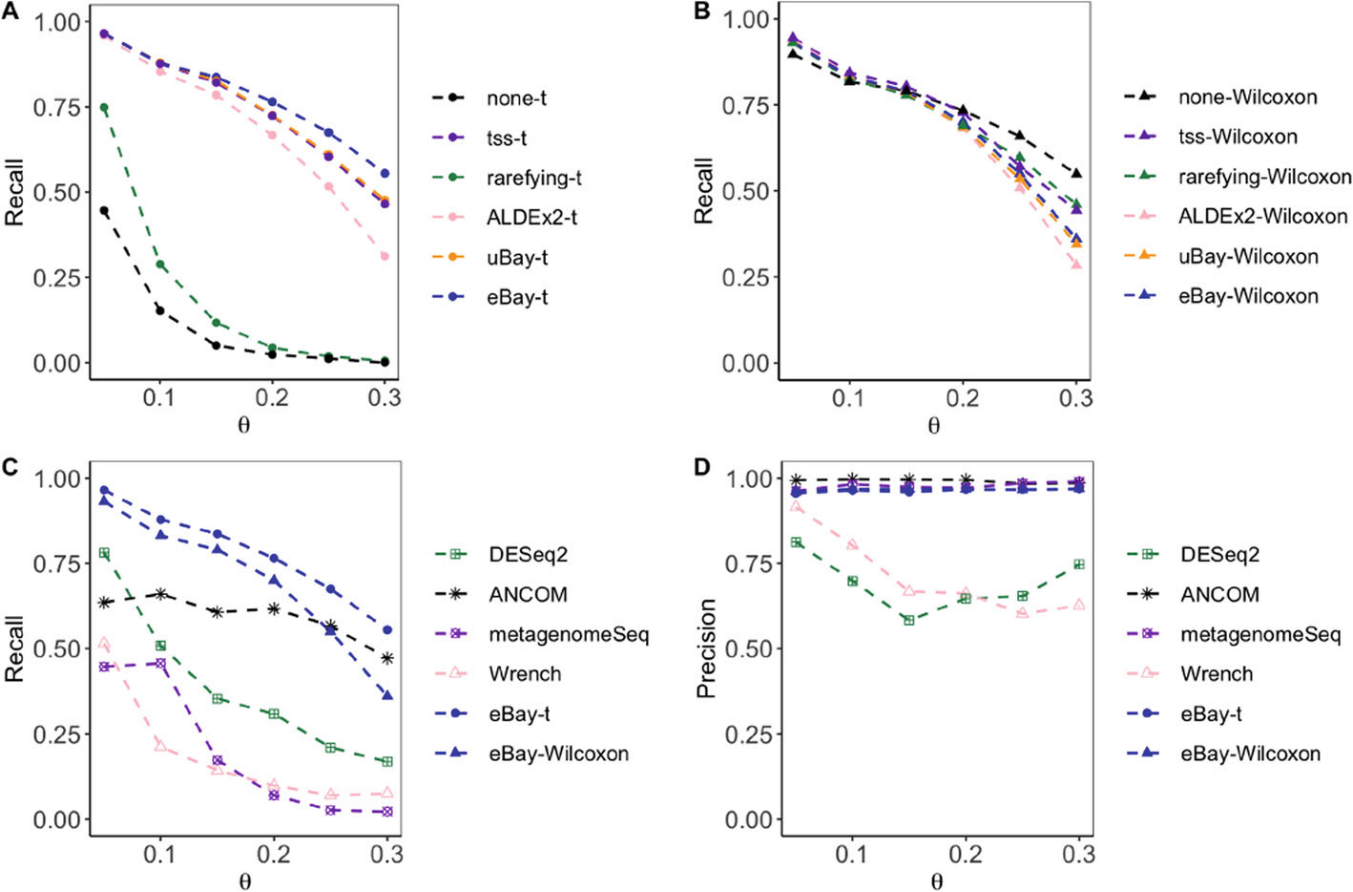
Comparison of recall and precision with data from DM across different *θ*. To detect differentially abundant taxa, we simulated 100 data sets from the DM model with *β*=0.25 and *θ*∈{0.05,0.1,0.15,0.2,0.25,0.3}. **a** and **b** Recall of t-test and Wilcoxon rank sum test with various normalization methods. **c** and **d** Recall and precision of DESeq2, ANCOM, metagenomeSeq, Wrench, and those of t-test and Wilcoxon rank sum test, both applied after counts were normalized by eBay

We estimated the recall and precision using 100 simulated data sets. The results are shown in Figs. 1 and 2. Generally, the recall and precision increased as the effect size *β* increased, and as the over-dispersion parameter *θ* decreased. From the upper panels we see that the recall of the empirical Bayes method, with t-test, was higher than other normalization methods. From the lower panels we can see that eBay-t and eBay-Wilcoxon were the overall winner. DESeq2, Wrench, metagenomeSeq had lower recall, and for small values of *β*, the precision of ANCOM was low.

### Simulations with tree information

**Fig. 3:**
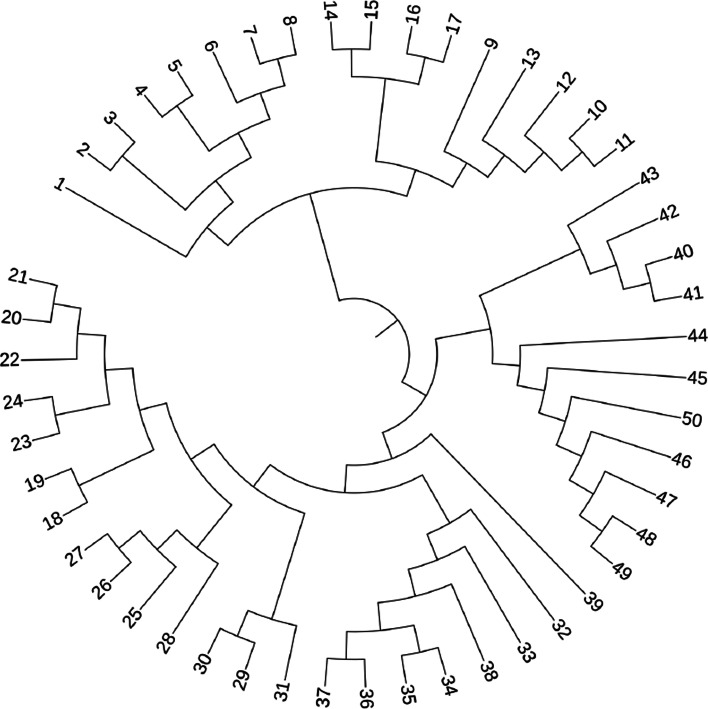
An example of a binary tree with 50 leaves and 49 internal nodes

In this section, taxa abundances were generated from the DTM model, with the tree structure shown in Fig. 3. We set *θ*=0.27 and *n*_1_=*n*_2_=50. The depth was sampled from a uniform distribution on (5000, 50000).

**The non-degenerate case.** A pair of nodes, labeled as 55 and 56, were set to be differentially abundant in a similar way as in the previous section. Specifically, at each tree split, we sampled *ϕ*_0_ from ***π***, and set ***ϕ***_1_=***ϕ***_2_=(*ϕ*_0_,1−*ϕ*_0_)^T^. We then increased the relative abundance of one of them, while decreasing the relative abundance of the other, by invoking (1) with *s*∈{55} and *t*∈{56} for *k*=1, and *s*∈{56} and *t*∈{55} for *k*=2. This led to 7 differentially abundant leaf nodes, labeled as 2–8 (Additional file 1: Figure S1). We set the effect size *β*∈{0.1,2,4,6,7,8}. We also used simulated data to investigate the effect of over-dispersion by fixing *β*=4 and setting *θ*∈{0.05,0.1,0.15,0.2,0.25,0.3}. Figures S2 and S3 summarize the simulation results. The empirical Bayes method eBay-tree was superior to other normalization methods, and when applied with t-test or Wilcoxon rank sum test, it outperformed DESeq2, ANCOM, Wrench, and metagenomeSeq.

**The degenerate case.** Two pairs of nodes, {55,56} and {57,58}, were set to be differentially abundant, but only 5 leaf nodes, labeled as 2, 3, 6, 7, 8, inherited the differences (Figure S4). This was achieved by taking *s*∈{55,57} and *t*∈{56,58} for *k*=1, and *s*∈{56,58} and *t*∈{55,57} for *k*=2. We set *β*∈{0.1,2,4,6,7,8}. On the other hand, we fixed *β*=4 and set *θ*∈ {0.05,0.1,0.15,0.2,0.25,0.3}. The simulation results were shown in Figures S5 and S6. Again, the conclusions were similar.

### More simulations

**Simulation from DM with a random tree.** We further examined the behavior of eBay-tree in the absence of tree, using the same data as in simulations without tree information. We generated the tree structure randomly, and used eBay-tree for data normalization. The results are summarized in Figure S7. We can see that incorporating the tree compulsorily did not deteriorate the performance much. In the presence of tree, we also compared the performance of the phylogeny-ware detection procedure and the global method of applying t-test or Wilcoxon rank sum test after normalizing data using (15). Figure S8 shows that the naive method failed.

**Fig. 4:**
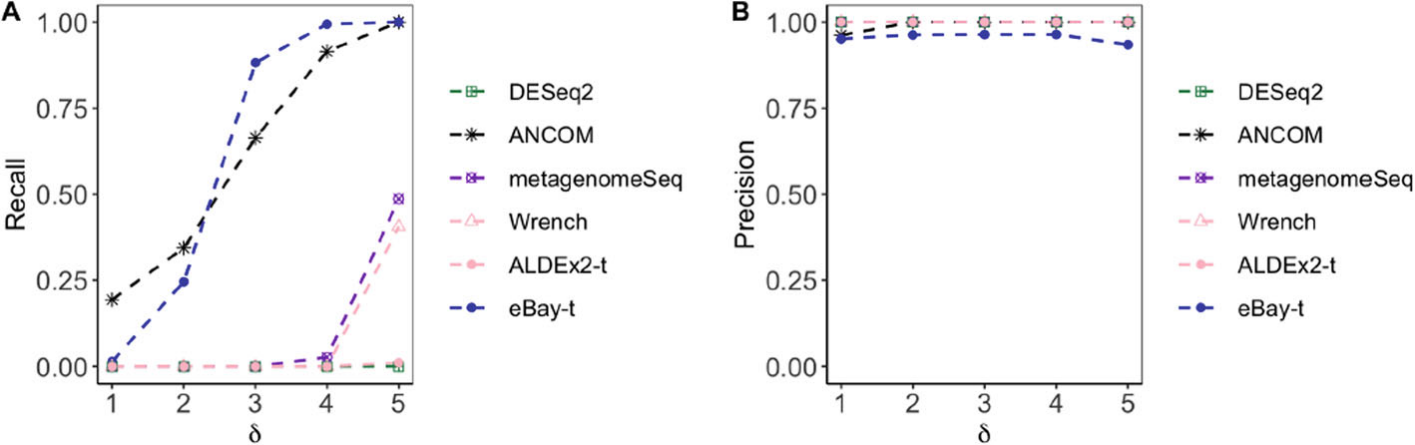
Comparison of recall and precision with data generated from the gamma-Poisson model across different *δ*. **a** and **b** Recall and precision of DESeq2, ANCOM, metagenomeSeq, Wrench, and that of t-test, which was applied after counts were normalized by ALDEx2 or eBay

**Simulated data from the gamma-Poisson model.** To assess the robustness of the proposed methodology, we generated taxa counts from the gamma-Poisson model which was used for evaluating the performance of ANCOM [14]. We set the sample size *n*_1_=20 for case and *n*_2_=30 for control with *p*=100. To generate the difference between two conditions, for the first 5 significant features in case, we changed the proportions of those features by adding *u*_*ij*_ to the Poisson parameter *μ*_*ij*_. For the remaining 5 features, we subtracted *u*_*ij*_ from the Poisson parameter *μ*_*ij*_. The *μ*_*ij*_ was sampling from a Gamma distribution *Gamma*(200,1) and *u*_*ij*_ was sampling from a uniform distribution *U*((*δ*−1)×30,*δ*×30) where *δ*∈{1,2,3,4,5}. The simulation results in Fig. 4 show that eBay-t compared favorably with ANCOM and both were superior to Wrench, DESeq2, and metagenomeSeq.

**Fig. 5:**
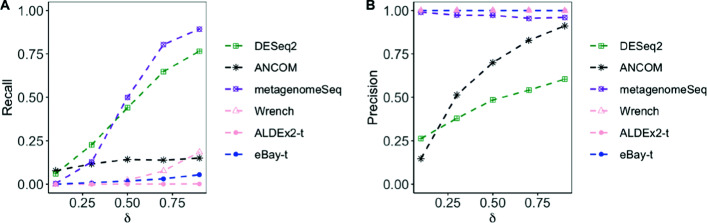
Comparison of recall and precision with data generated from the zero-inflated log-normal model across different *δ*. **a** and **b** Recall and precision of DESeq2, ANCOM, metagenomeSeq, Wrench, and that of t-test, which was applied after counts were normalized by ALDEx2 or eBay

**Simulated data from the zero-inflated log-normal (ZILN) model.** As suggested by a referee, we also generated taxa counts from the zero-inflated log-normal model which was used for assessing the performance of metagenomeSeq [8]. We set the sample size *n*_1_=*n*_2_=50 and the number of taxa *p*=100. To generate the difference between conditions, for the first 5 significant features in one of the conditions, we changed the proportions of those features by adding 1/50×*δ* percentage of the sample’s total counts. For the remaining 5 features, we subtracted 1/50×*δ* percentage of the sample’s total counts. The *δ* was set to be {0.1,0.3,0.5,0.7,0.9}. As expected, we see from Fig. 5 that metagenomeSeq outperformed DESeq2. Unfortunately, methods treating microbiome data

as compositions, especially eBay and ALDEx2 in the Bayesian framework, failed in this case. The reason is that the zeros generated by the ZILN model are all structural zeros, while in eBay and ALDEx2 it is assumed implicitly that zeros are the result of under-sampling. As will be discussed later, extending the empirical Bayes method to handling both structural zeros and sampling zeros is interesting and important.

Finally, in Figure S9, we show comparative timings in seconds and space in bytes for problems with *n*_1_=*n*_2_=50 and different numbers of taxa. While eBay was computationally more efficient, with parallel computation the computational complexity of eBay-tree performed similarly with eBay.

### Gut microbiota and malnutrition

Childhood undernutrition is a significant health problem in Southern Asia and sub Saharan Africa, and severe acute malnutrition (SAM) remains a major cause of child mortality worldwide [33]. For this reason, the World Health Organization updated guidelines for the improved management of SAM in infants and children [34]. In a recent study of 996 stool samples collected monthly from 50 healthy Bangladeshi children during the first 2 years of life, [32] identified bacterial taxonomic biomarkers for characterizing gut-microbiota maturation. By applying random forests from the perspective of regression, they determined a list of 60 bacterial species, ranked in descending order of their importance to the regression. Incorporating these biomarkers into a prediction model, and applying this model to children with SAM enrolled in a randomized trial, they showed that SAM is significantly associated with microbiota immaturity.

Rather than summarizing the relative abundances of these 60 bacterial taxa into a single index (i.e., the predicted value), we revisited the problem in terms of differential abundance testing. To eliminate the effect of age, we restricted our analysis to 12 to 18-month-old children. There were 20 healthy children in the singleton validation dataset and 27 children with SAM. We further filtered bacterial species with prevalance less than 20%, resulting in 50 taxa. We extracted representative sequences for these taxa, performed sequence alignment, and then constructed a phylogenetic tree (Figure S10), using the default and recommended methods PyNAST and FastTree in QIIME [12]. We applied t-test and Wilcoxon rank sum test after normalizing counts by the tree-based empirical Bayes method and other methods in Table 1, and compared them to DESeq2, ANCOM, Wrench, and metagenomeSeq. Note that eBay took 0.41 seconds to analyze the data on a Macbook Pro (Intel Corei5, 1.4 GHz, 8GB RAM).

**Table 1:** Normalization methods

Method	Description
none	Raw counts are not transformed.
tss	Total sum scaling. Raw counts are divided by the library size.
css	Cumulative sum scaling. As above, except that for each sample a quantile is calculated and the total sum is replaced by the sum up to and including that quantile.
rarefying	Each observation is subsampled to even depth. This method is implemented in the R package phyloseq [35]. We use the function rarefy_even_depth with sample.size=0.90*min(sample.size).
uBay	A standard Bayesian method that infers the posterior distribution of proportions as the product of the multinomial likelihood with a Dirichlet prior. Following [24], we set ***α***=(1/2,…,1/2)^T^ and convert raw counts to proportions by (6).
ALDEx2	A Bayesian method that infers the posterior distribution of proportions in the same way as uBay. However, rather than using the posterior mean, Monte–Carlo draws from the posterior distribution are used in downstream analysis [36].
eBay	The same as uBay, except that hyper-parameters of the Dirichlet prior are estimated from data by maximizing the marginal likelihood. We use the proposed empirical Bayes formula (9).
eBay-tree	The tree-based extension (15) of eBay.

**Fig. 6:**
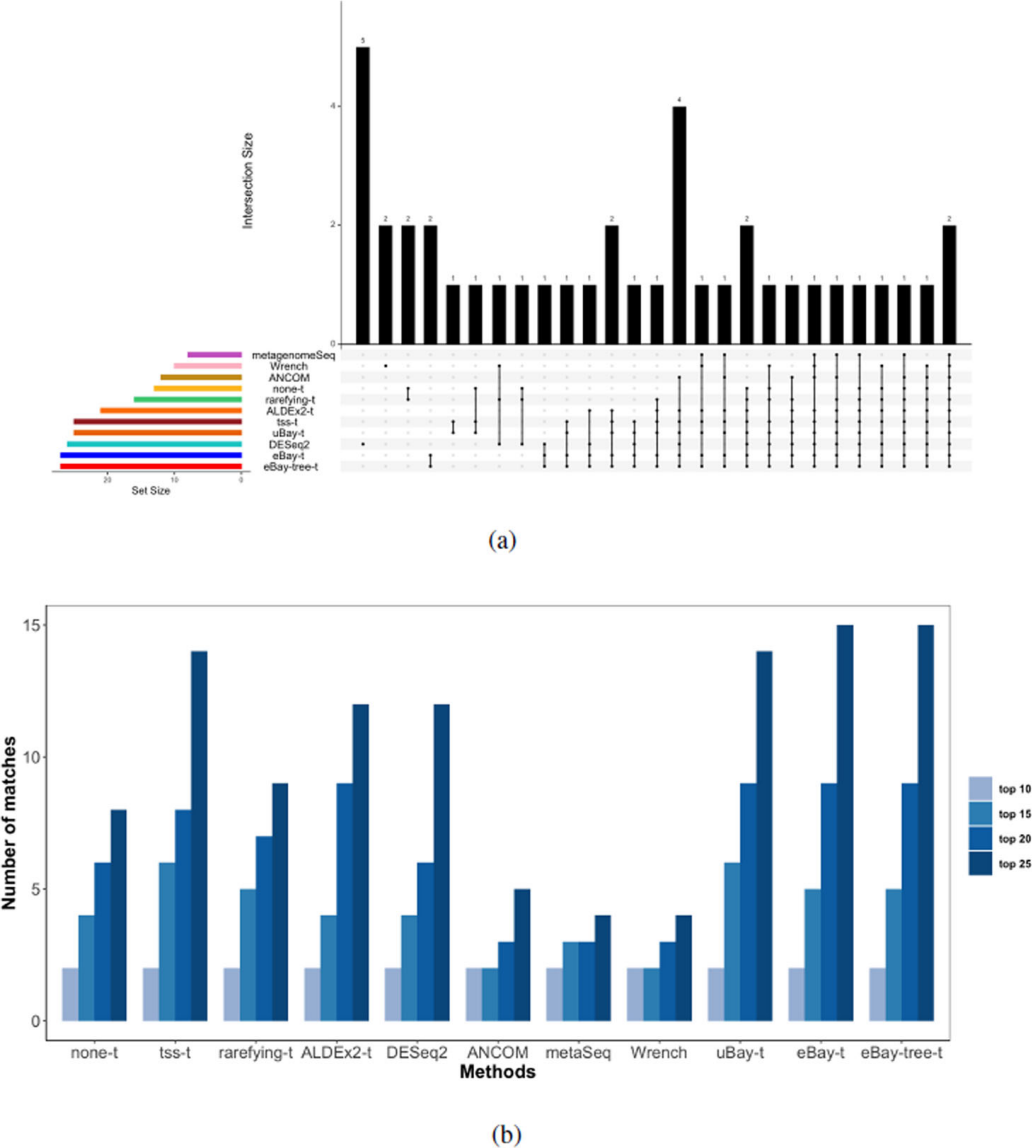
Differentially abundant bacterial species between healthy children and children with SAM. **a** Visualization of set intersections among differential abundance testing methods in Table 2. **b** The number of matches between the top *K* taxa identified by random forests and the top *K* differentially abundant taxa detected by various testing methods. metaSeq: metagenomeSeq

To assess the performance of our method and other methods, we recorded the lists of differentially abundant taxa. In addition, for each method, we ordered the taxa according to their p-values, and calculated the number of matches between the top *K* differentially abundant taxa and the top *K* taxa in the ranked list of 60 bacterial species, where *K*=10,15,20, and 25. The results are summarized in Fig. 6 and Figure S11. From Fig. 6a, we can see that eBay-t and eBay-tree-t detected more differentially abundant species than other methods. The two taxa detected uniquely by eBay-t and eBay-tree-t were *Rumicnococcus*_*sp*_5_1_39*BFAA* and *Megamonas*. Million et al. [37] indicated that *Ruminococcus*_*sp*_5_1_39*BFAA* tends to be depleted in malnourished children, while *Megamonas* was reported to be significantly altered in the malnourished children compared to age-matched healthy children [32]. Furthermore, Fig. 6b shows that the ranked list of taxa detected by eBay-t and eBay-tree-t was more concordant with that identified by the random forests algorithm. FastTree infers the phylogeny by maximum likelihood. Alternatively, we computed the distances between any two species based on an evolution model [38], and then built a phylogenetic tree (Figure S12) based on these distances [39]. The corresponding results are summarized in Figures S13 and S14, and the conclusions are qualitatively similar. These results confirm that compared to healthy children, children with SAM had significant gut-microbiota immaturity.

**Table 2:** Differential abundance testing methods

Method	Description
t-test	Welch two sample t-test. We use the R built-in t.test function with default parameters. This test applies to either raw counts or transformed data.
Wilcoxon	Wilcoxon rank-sum test. We use the R built-in function wilcox.test with default parameters. This test applies to either raw counts or transformed data.
DESeq2	A popular method from the field of RNA-seq. It is based on a negative binomial model for raw counts, and is implemented in R package DESeq2 [50]. We use the built-in library size normalization and default parameters.
ANCOM	A novel method for detecting differentially abundant taxa at the ecosystem level using the specimen level relative abundance data. This test is implemented in the R package ancom.R [14]. We use the default setting.
metagenomeSeq	As with ANCOM, this method is developed specifically for microbial datasets. It is based on a zero-inflated Gaussian mixture model for log read counts. We use the function fitFeatureModel in the R package metagenomeSeq [51], with cumulative sum scaling and default parameters.
Wrench	A new technique for compositional bias correction in sparse sequencing count data [20]. It fits a negative binomial log-linear model for reference-based data normalization, and then runs a likelihood ratio test for detecting differentially abundant taxa. We use the functions glmFit and glmLRT in the R package edgeR [52].

### Gut microbiome and body mass index

**Fig. 7:**
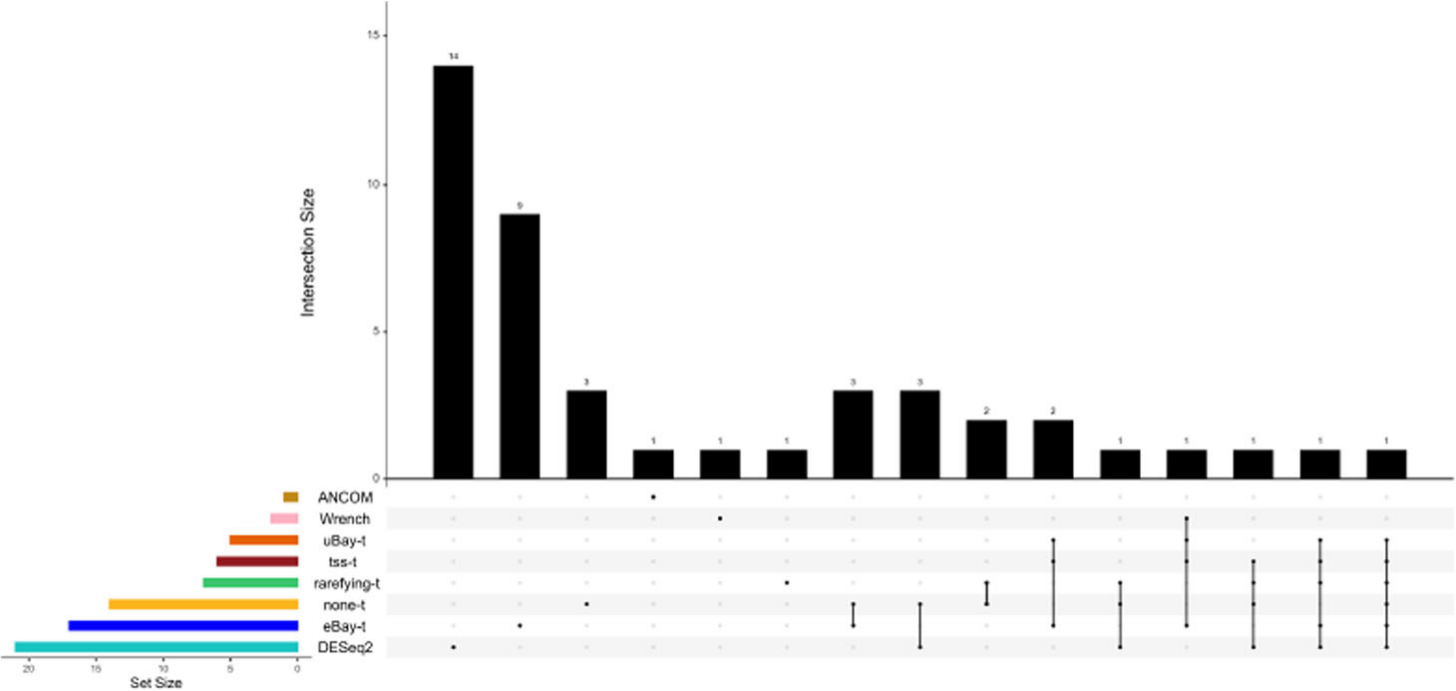
Differentially abundant bacterial species between normal weight and obese individuals. Visualization of set intersections among differential abundance testing methods in Table 2

Studies have shown that gut microbiome is associated with body mass index (BMI) and explains a significant fraction of BMI variation [5]. In a study of the impact of long-term dietary patterns on gut microbiome composition, [40] showed that taxa correlated with BMI also correlated with fat and percent calories from saturated fatty acids. In this study, the researchers enrolled 98 healthy volunteers and collected their stool samples as well as diet information. DNA samples were extracted and analyzed by 454/Roche pyrosequencing, and sequence reads were processed by the QIIME pipeline. To explore the relationship between BMI and gut microbiota, we reanalysed the data via differential abundance testing. Following the World Health Organization guideline, we categorized BMI as normal weight, overweight, and obese, and for simplicity we focused on the normal weight and obese individuals. After filtering the taxa with prevalence less than 10% and abundance <0.2% in all samples, we were left with 314 taxa and 70 samples. eBay took 1.678 seconds to process the data on a Macbook Pro (Intel Corei5, 1.4 GHz, 8GB RAM). The results are summarized in Fig. 7. The 9 taxa identified uniquely by eBay-t were mainly from the families *Lachnospiraceae* and *Ruminococcaceae*, both of which were reported to be significantly correlated with BMI [41, 42].

## Discussion

Although the important role of microbiota in human health and disease has been recognized increasingly over the past decade, data from high-throughput DNA sequencing present challenges to statistical analysis and interpretation. We have proposed an empirical Bayes technique for microbiome data normalization prior to downstream analysis. Assuming a multinomial distribution for the read counts and specifying a Dirichlet prior for the underlying proportions, our method shrinks the relative abundances towards the mean vector of the prior. The marginal distribution of the data allows for over-dispersion and has the same set of parameters as the prior distribution. We estimated these parameters empirically from the data by maximizing the evidence. To incorporate the phylogenetic tree in the normalization process, we extended our method by taking as the prior a product of Dirichlet distributions that factorized over the tree. We examined the downstream effect of normalization in the context of differential abundance analysis, by applying t-test and Wilcoxon rank sum test to the normalized data. In the presence of tree, rather than using the normalized data directly, we proposed a phylogeny-aware differential abundance detection procedure by carrying out local tests at tree splits.

The excessive number of zeros in bacterial counts can lead to some inefficiency in the normalization and downstream analysis. In this paper, we have introduced an empirical Bayes method to normalize data and we assume implicitly that all microbes are present in the microbial ecosystem and the zeros are the result of undersampling. However, in the presence of hundreds or thousands of bacterial species, these zeros can also represent components that are truly absent from the community [8, 9], especially when the specimens are drawn from different environments. How to normalize count data that allows zero-inflation is an interesting research topic. The zero-inflated generalized Dirichlet model [43] can potentially provide a solution to this problem. Work along this line is in progress.

## Conclusions

Uneven library size, data sparsity, compositionality, and over-dispersion, all make drawing valid biological inferences from microbial datasets difficult. To overcome these challenges, we proposed an empirical Bayes technique for microbiome data normalization prior to downstream analysis. We further extended our method by incorporating the phylogenetic tree into the normalization process. We examined the downstream effect of normalization in the context of differential abundance analysis. In the presence of tree, we proposed a phylogeny-aware detection procedure. Results from an extensive simulation study and real data applications showed that the empirical Bayes approach was more efficient than other normalization methods, and the corresponding testing method compared favorably with state-of-the-art methods.

## Methods

Consider a microbiome dataset with *n* samples and *p* OTUs. For the *i*th sample, let ***x***_*i*_=(*x*_*i*1_,...,*x*_*ip*_)^T^ denote the vector of read counts of *p* OTUs, and $N_{i}=\sum _{j=1}^{p} x_{ij}$ the total number of reads. Total sum scaling can be derived through maximum likelihood. Given *N*_*i*_, it is natural to model the abundance vector according to a multinomial distribution, ***x***_*i*_∼*Mult*(***π***_*i*_;*N*_*i*_). The probability mass function is

2 $$\begin{array}{@{}rcl@{}} f_{Mult}(\boldsymbol{x}_{i}; \boldsymbol{\pi}_{i}, N_{i}) = \frac{\Gamma(N_{i}+1)}{\prod_{j=1}^{p}\Gamma(x_{ij}+1)}\prod_{j=1}^{p}\pi_{ij}^{x_{ij}}, \end{array} $$

where $\boldsymbol {\pi }_{i} = (\pi _{i1},...,\pi _{ip})^{\mathrm {T}}, 0<\pi _{ij}<1, \sum _{j=1}^{p}\pi _{ij} = 1$, and *Γ*(·) is the gamma function. Then the method of maximum likelihood yields the naive count normalization

3 $$ \tilde{\pi}_{ij} = \frac{x_{ij}}{N_{i}}.  $$

### Empirical Bayes normalization

One disadvantage of total sum scaling is that the estimates for OTUs with zero counts are simply zero, causing difficulty in downstream analyses, such as log-ratio based compositional data analysis. To overcome this problem, we consider a Bayesian approach. Specifically, we assume that ***x***_*i*_∼*Mult*(***π***_*i*_;*N*_*i*_), and specify a prior distribution for ***π***_*i*_. We then calculate the posterior for ***π***_*i*_ given ***x***_*i*_, and compute the posterior mean estimate.

The most common and convenient prior for ***π***_*i*_ is the Dirichlet distribution [44]. This distribution, denoted by *Dir*(***α***), is parameterized by a *p*-vector of positive scalars, ***α***=(*α*_1_,…,*α*_*p*_)^T^, and has probability density function

4 $$ f_{Dir}(\boldsymbol\pi_{i};\boldsymbol\alpha)=\frac{\Gamma(\sum_{j=1}^{p}\alpha_{j})}{\prod_{j=1}^{p}\Gamma(\alpha_{j})}\prod_{j=1}^{p}\pi_{ij}^{\alpha_{j}-1}.  $$

Multiplying the multinomial distribution *Mult*(***π***_*i*_;*N*_*i*_) by the Dirichlet prior *Dir*(***α***) gives the posterior distribution

5 $$ \begin{aligned} &f(\boldsymbol \pi|\boldsymbol x_{i},\boldsymbol\alpha) = \frac{\Gamma(N_{i}+\sum_{j=1}^{p}\alpha_{j})}{\prod_{j=1}^{p}\Gamma(x_{ij}+\alpha_{j})}\prod_{j=1}^{p}\pi_{j}^{x_{ij}+\alpha_{j}-1}.\\ \end{aligned}  $$

This is the density of *Dir*(***x***_*i*_+***α***). The posterior mean is given by

6 $$ \begin{aligned} E(\pi_{ij}|\boldsymbol x_{i},\boldsymbol\alpha)=\frac{x_{ij}+\alpha_{j}}{\sum_{j=1}^{p}(x_{ij}+\alpha_{j})}. \end{aligned}  $$

Posterior Bayesian estimation produces non-zero estimates for the true proportions. Furthermore, it is easy to check that the posterior mean is a weighted average of the vector of raw proportions and the mean of the prior distribution:

7 $$ \begin{aligned} E(\pi_{ij}|\boldsymbol x_{i},\boldsymbol\alpha)=\frac{N_{i}}{N_{i}+\alpha_{+}}\tilde{\pi}_{ij}+\frac{\alpha_{+}}{N_{i}+\alpha_{+}}\phi_{j}, \end{aligned}  $$

where $\phi _{j} = \alpha _{j}/\sum _{j = 1}^{p}\alpha _{j}$ and $\alpha _{+} = \sum _{j = 1}^{p}\alpha _{j}$. Put another way, we shrink the maximum likelihood estimates towards our knowledge about ***π***_*i*_ before we see the data.

In practice, the hyper-parameters *α*_*j*_ are unknown, and so we cannot use posterior Bayesian estimates. Uniform priors, which assume that *α*_1_=⋯=*α*_*p*_, are used in [24] and [25]. The mean vector of a uniform prior, (1/*p*,...,1/*p*)^T^, is the center or neural element of the (*p*−1)-dimensional simplex with the Aitchison metric [16]. Nevertheless, we do not have to take this composition as the preferred shrinking point. In the rest of this section, we propose an empirical Bayes approach by empirically estimating *α*_*j*_ from the data.

Note that after integrating ***x***_*i*_∼*Mult*(***π***_*i*_;*N*_*i*_) over ***π***_*i*_∼*Dir*(***α***), the marginal distribution of ***x***_*i*_ is Dirichlet-multinomial, ***x***_*i*_∼*DirMulti*(***α***), with probability mass function

8 $$\begin{array}{@{}rcl@{}} &&f_{DM}(\boldsymbol x_{i}|\boldsymbol \alpha)= \frac{\Gamma(N_{i}+1)\Gamma(\alpha_{+}) }{\Gamma(N_{i}+\alpha_{+})} \prod_{j=1}^{p}\frac{\Gamma(\alpha_{j}+x_{ij})}{\Gamma(x_{ij}+1)\Gamma(\alpha_{j})}. \end{array} $$

The DM distribution has the same set of parameters as the Dirichlet prior. Furthermore, it is the most common distribution for modeling over-dispersed and multivariate taxa count data [28, 45]. Let *θ*=1/(1+*α*_+_), we call *θ* the over-dispersion parameter. Let ${\hat {\boldsymbol \alpha }}$ be the maximum likelihood estimate. Substituting it into (6) gives the empirical Bayes solution for normalization

9 $$\begin{array}{@{}rcl@{}} &&E(\pi_{ij}|\boldsymbol x_{i}, \hat{{\boldsymbol\alpha}})=\frac{x_{ij}+\hat{\alpha}_{j}}{\sum_{j=1}^{p}(x_{ij}+\hat{\alpha}_{j})}. \end{array} $$

### Phylogeny-aware normalization

Suppose that the phylogenetic relationships among OTUs can be encoded by a rooted tree $\mathcal {T} = (\mathcal {L}, \mathcal {I})$, where terminal nodes, or leaves, in $\mathcal {L}$ correspond to OTUs, and internal nodes in $\mathcal {I}$ represent bacterial taxa at different taxonomic levels. Figure 3 shows an example of a binary tree over 50 OTUs. For each internal node $A \in \mathcal {I}$, let $\mathcal {C}(A)$ be the set of child nodes of *A*. For each *A* and $w \in \mathcal {C}(A)$, let *x*_*Aw*_ and *π*_*Aw*_ be the total count and probability in the branch from *A* to *w*. Here, for ease of notation, we omit the subscript *i*. One attractive property of the multinomial distribution is that it can be factorized over $\mathcal {T}$ [29]. Specifically, let $ b_{Aw} = \pi _{Aw} / \sum _{w \in \mathcal {C}(A)}\pi _{Aw}$, ***b***_*A*_=(*b*_*Aw*_,*w*∈*A*), and ***x***_*A*_=(*x*_*Aw*_,*w*∈*A*), then

10 $$\begin{array}{@{}rcl@{}} f_{MN}(x_{i};\boldsymbol \pi_{i},N_{i})&=&\prod_{A\in \mathcal{I}}f_{MN}(\boldsymbol{x}_{A};\boldsymbol{b}_{A}) \\ &=&\prod_{A\in \mathcal{I}}\frac{\Gamma(\sum_{w\in \mathcal{C}(A)}x_{Aw}+1)}{\prod_{w \in \mathcal{C}(A)}\Gamma(x_{Aw}+1)}\prod_{w \in \mathcal{C}(A)}b_{Aw}^{x_{Aw}}. \end{array} $$

The conjugate prior for this parameterization is no longer a single global Dirichlet density, but rather a product of local Dirichlet densities, one for each internal node:

11 $$\begin{array}{@{}rcl@{}} \prod_{A \in \mathcal{I}}f_{D}(\boldsymbol{\pi}_{A};\boldsymbol{\alpha}_{A})=\prod_{A\in \mathcal{I}}\frac{\Gamma(\sum_{w\in \mathcal{C}(A)}\alpha_{Aw})}{\prod_{w \in \mathcal{C}(A)}\Gamma(\alpha_{Aw})}\prod_{w \in \mathcal{C}(A)}\pi_{Aw}^{\alpha_{Aw}-1}. \end{array} $$

This is known as the Dirichlet-tree distribution [46]. The posterior distribution has the form

12 $$\begin{array}{@{}rcl@{}} \prod_{A\in \mathcal{I}} f(\boldsymbol \pi_{A}| \boldsymbol x_{A}, \boldsymbol\alpha_{A})=\prod_{A \in \mathcal{I}}\frac{\Gamma\{\sum_{w \in \mathcal{C}(A)}(x_{Aw}+\alpha_{Aw})\}}{\prod_{w \in \mathcal{C}(A)}\Gamma(x_{Aw}+\alpha_{Aw})}\prod_{w \in \mathcal{C}(A)}\pi_{Aw}^{x_{Aw}+\alpha_{Aw}-1}. \end{array} $$

This density is exactly that of a Dirichlet-tree distribution, except that we update the hyper-parameters after seeing the data.

The development so far is based on Dirichlet priors on branches. The posterior density function of ***π*** given the data can be computed by a change of variables and is given in [47]. Furthermore, the posterior mean of ***π*** is

13 $$\begin{array}{@{}rcl@{}} &&E(\pi_{l}\mid \boldsymbol{x},\boldsymbol \alpha_{A}, A\in \mathcal{I}) =\prod_{A\in \mathcal{I}}\prod_{w \in \mathcal{C}(A)}\left\{\frac {x_{Aw}+\alpha_{Aw}}{\sum_{w \in \mathcal{C}(A)}(x_{Aw}+\alpha_{Aw})}\right\}^{\delta_{Aw}(l)}, \end{array} $$

where we define *δ*_*Aw*_(*l*) to be 1, if the branch from *A* to *w* leads to $l \in \mathcal {L}$, and 0 otherwise.

The remaining step is the same: the Bayes estimator is itself being empirically estimated from the data by maximizing the evidence, i.e., the marginal distribution of the data. This distribution, known as the Dirichlet-tree multinomial distribution (DTM), is a product of DM distributions that factorize over the tree

14 $$\begin{array}{@{}rcl@{}} &&f_{DTM}(\boldsymbol x,\boldsymbol{\alpha}_{A}, A \in \mathcal{I}) \\ &=&\prod_{A \in \mathcal{I}} \frac{\Gamma(\sum_{w \in \mathcal{C}(A)}x_{Aw}+1)\Gamma(\sum_{w \in \mathcal{C}(A)}\alpha_{Aw})}{\Gamma\{\sum_{w \in \mathcal{C}(A)}(x_{Aw}+\alpha_{Aw})\}}\prod_{w \in \mathcal{C}(A)}\frac {\Gamma(x_{Aw}+\alpha_{Aw})}{\Gamma (x_{Aw}+1)\Gamma(\alpha_{Aw})}. \\ \end{array} $$

Comparing to DM, a distinctive property of DTM is that the correlations between bacterial counts can be simultaneously negative and positive [29, 31]. Since the distributions placed on different internal nodes are independent, maximum likelihood estimation can be carried out separately and in parallel. Let $\hat {\boldsymbol {\alpha }}_{A}$ be the maximum likelihood estimate. Substituting it into (13) leads to the phylogeny-aware normalization

15 $$\begin{array}{@{}rcl@{}} &&E(\pi_{l}\mid \boldsymbol{x}, \hat{\boldsymbol \alpha}_{A}, A\in \mathcal{I})=\prod_{A\in \mathcal{I}}\prod_{w \in \mathcal{C}(A)}\left\{\frac {x_{Aw}+\hat{\alpha}_{Aw}}{\sum_{w \in \mathcal{C}(A)}(x_{Aw}+\hat{\alpha}_{Aw})}\right\}^{\delta_{Aw}(l)}. \end{array} $$

### Centered log-ratio transformation

The normalization methods investigated in this paper are shown in Table 1. Except for rarefying, all methods infer proportions from the raw read counts. Because proportions are constrained by the simplex, standard statistical methods for downstream analyses are not applicable. To convert proportions into linear independent components, [48] introduced the centered log-ratio transformation, which is an isometric transformation of the simplex with the Aitchison metric onto a subspace of real space with the Euclidean metric. Let (*u*_1_,…,*u*_*m*_)^T^ denote a generic *m*-vector of proportions. This transformation has the form

$$v_{j}=\log(u_{j})-\frac{\sum_{k=1}^{m}\log(u_{k})}{m}.$$

Transformed data are then analyzed in the same way as standard data. We employ this strategy in this paper.

### Differential abundance analysis

After effective normalization, a common downstream analysis is differential abundance testing. In this section, we examine the impact of normalization using the results from a differential abundance analysis. As with [10, 11], and [49], we focus on detecting microbes that are differentially abundant between two conditions. Table 2 lists the methods considered in this paper. For the moment, we assume that the tree information is not available. Among these, t-test and Wilcoxon rank sum test are standard methods for comparing two groups, DESeq2 [50] is model-based and is borrowed from the field of RNA-seq, and metagenomeSeq [8] and ANCOM [14] are also model-based and are proposed specifically for microbiome sequencing data.

Note that t-test and Wilcoxon rank sum test apply to either raw counts or proportions, and ANCOM normalizes the raw counts by taking ratios relative to a reference taxon. DESeq2 and metagenomeSeq use raw counts, but each of them has a built-in normalization process. Furthermore, ANCOM involves the replacement of zeros by a small positive number. For simplicity, a pseudocount of one is added to the raw counts before applying normalization. To control the false discovery rate (FDR), all tests are corrected for multiple testing using the Benjamini–Hochberg procedure [53].

Differential abundance analysis in the presence of tree structure is somewhat complicated. To our knowledge, incorporating the dependence structure among the microbes into any of ANCOM, DESeq2, and metagenomeSeq is not trivial and deserves further study. Here, we propose a phylogeny-aware detection approach based on either t-test or Wilcoxon rank sum test. One simple approach is to do the test directly after the tree-based normalization (15). However, the obvious drawback of this naive approach is that the estimation error in a tree split is propagated down to all of the splits below it. To alleviate this problem, instead of a global test, we carry out local tests at tree splits. If a node is differentially abundant, then so are all of its descendants. Since the number of nodes at a split is much lower than the number of leaf nodes and the local tests can be done split-by-split, this approach is computationally more stable and less intensive.

There is an exception. If two nodes are differentially abundant and are both ancestors of a leaf node, then it is possible that the leaf node is not differentially abundant. To finesse the problem, we note that in these degenerate cases, there must be a path from the differentially abundant nodes to the leaf node. We make a correction by locating the most recent ancestor node to this path that is non-differentially abundant, and do test on the set of all leaf nodes of this node and update the results. The tree-guided detection procedure is summarized in **Algorithm 1**.


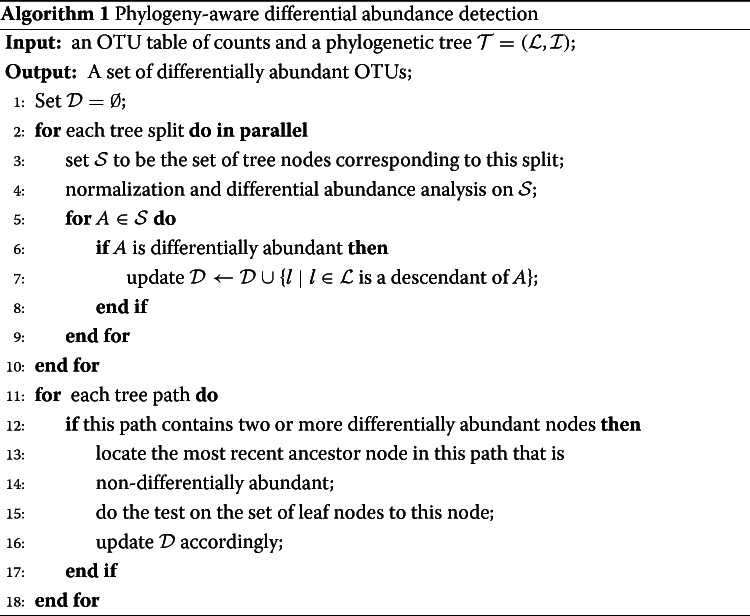


## Supplementary information

**Additional file 1** Figure S1. A phylogenetic tree representing the non-degenerate case. Figure S2. Comparison of recall and precision with data generated from the DTM model across different *β*: the non-degenerate case. Figure S3. Comparison of recall and precision with data generated from the DTM model across different *θ*: the non-degenerate case. Figure S4. A phylogenetic tree representing the degenerate case. Figure S5. Comparison of recall and precision with data generated from the DTM model across different *β*: the degenerate case. Figure S6. Comparison of recall and precision with data generated from the DTM model across different *θ*: the degenerate case. Figure S7. Comparison of recall and precision between eBay and eBay-tree. Figure S8. Comparison of recall and precision between eBay-tree and eBay-tree (global). Figure S9. Timings (seconds) and space (log(bytes)), averaged over 10 runs with data generated from the DTM model with *n*_1_=*n*_2_=50, versus the number of taxa. Figure S10. The phylogenetic tree of 50 bacterial taxa inferred by maximum likelihood. Figure S11. Differentially abundant species detected by Wilcoxon rank sum test based on the tree in Figure S10. Figure S12. The phylogenetic tree of 50 bacterial taxa built based on distances. Figure S13. Differentially abundant species detected by t-test based on the tree in Figure S12. Figure S14. Differentially abundant species detected by Wilcoxon rank sum test based on the tree in Figure S12.

## Abbreviations

OTUs: Operational taxonomic units
DM: Dirichlet-multinomial
DTM: Dirichlet-tree multinomial
FDR: False discovery rate
SAM: Severe acute malnutrition
ZILN: Zero-inflated log-normal
