# Supplementary material for: An empirical Bayes approach to normalization and differential abundance testing for microbiome data
Source: BMC Bioinformatics. 2020 Jun 3;21:225. doi: 10.1186/s12859-020-03552-z (PMC7268703; doi:10.1186/s12859-020-03552-z)
Supplement: Supplementary file 1 — Additional file 1 Figure S1. A phylogenetic tree representing the non-degenerate case. Figure S2. Comparison of recall and precision with data generated from the DTM model across different β: the non-degenerate case. Figure S3. Comparison of recall and precision with data generated from the DTM model across different θ: the non-degenerate case. Figure S4. A phylogenetic tree representing the degenerate case. Figure S5. Comparison of recall and precision with data generated from the DTM model across different β: the degenerate case. Figure S6. Comparison of recall and precision with data generated from the DTM model across different θ: the degenerate case. Figure S7. Comparison of recall and precision between eBay and eBay-tree. Figure S8. Comparison of recall and precision between eBay-tree and eBay-tree (global). Figure S9. Timings (seconds) and space (log(bytes)), averaged over 10 runs with data generated from the DTM model with n1=n2=50, versus the number of taxa. Figure S10. The phylogenetic tree of 50 bacterial taxa inferred by maximum likelihood. Figure S11. Differentially abundant species detected by Wilcoxon rank sum test based on the tree in Figure S10. Figure S12. The phylogenetic tree of 50 bacterial taxa built based on distances. Figure S13. Differentially abundant species detected by t-test based on the tree in Figure S12. Figure S14. Differentially abundant species detected by Wilcoxon rank sum test based on the tree in Figure S12. [file 12859_2020_3552_MOESM1_ESM.pdf]

Supplementary materials for
An empirical Bayes approach to normalization and differential abundance testing for microbiome data

Tiantian Liu[1,3], Hongyu Zhao[2,3], and Tao Wang[1,3,4,*]

[1]Department of Bioinformatics and Biostatistics, Shanghai Jiao Tong University
[2]Department of Biostatistics, Yale University
[3]SJTU-Yale Joint Center for Biostatistics and Data Science, Shanghai Jiao Tong University
[4]MoE Key Lab of Artificial Intelligence, Shanghai Jiao Tong University
[*]Corresponding author: neowangtao@sjtu.edu.cn

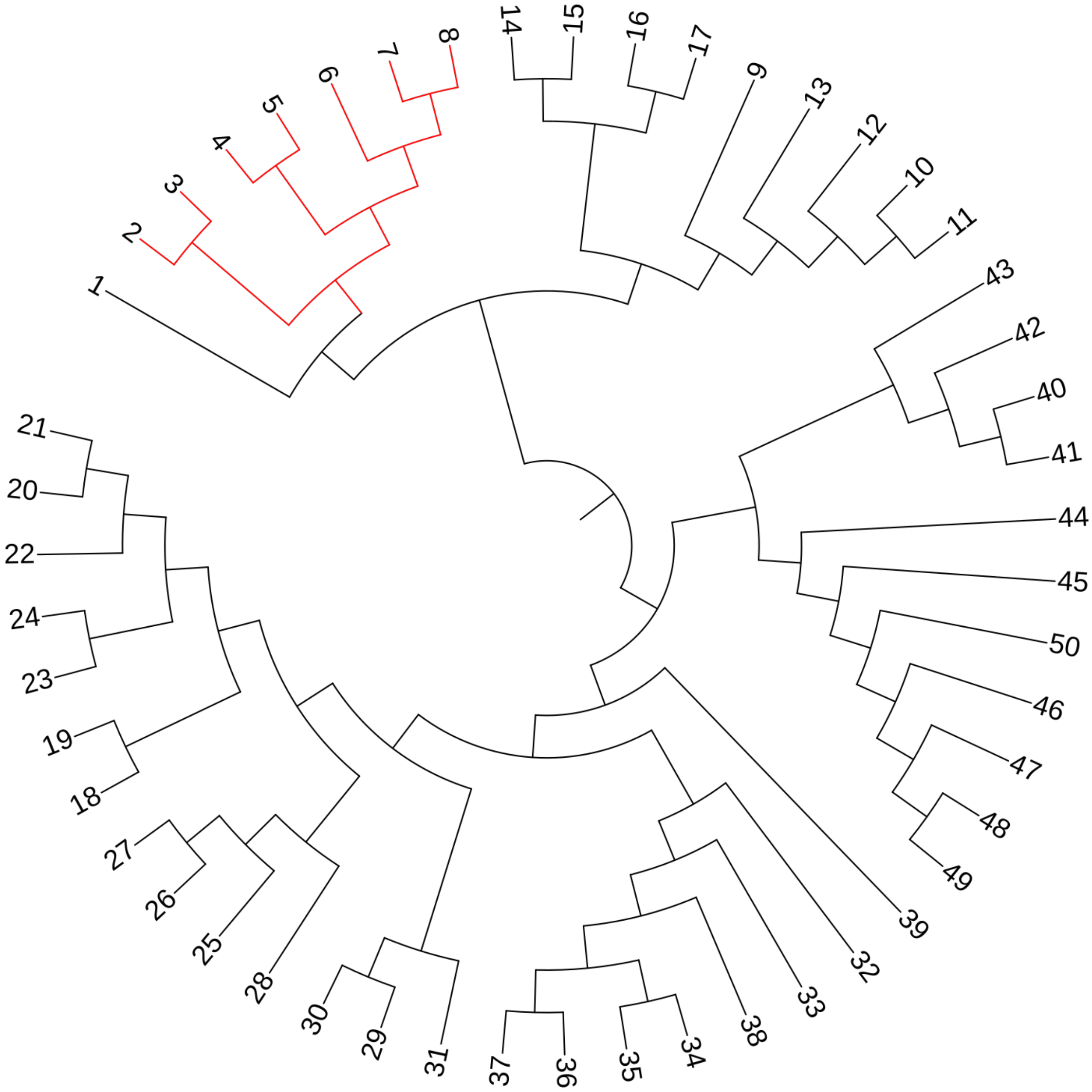

Figure S1 The non-degenerate case. A pair of nodes, labeled as 55 and 56, were set to be differentially abundant. This led to 7 differentially abundant leaf nodes, labeled as 2-8.

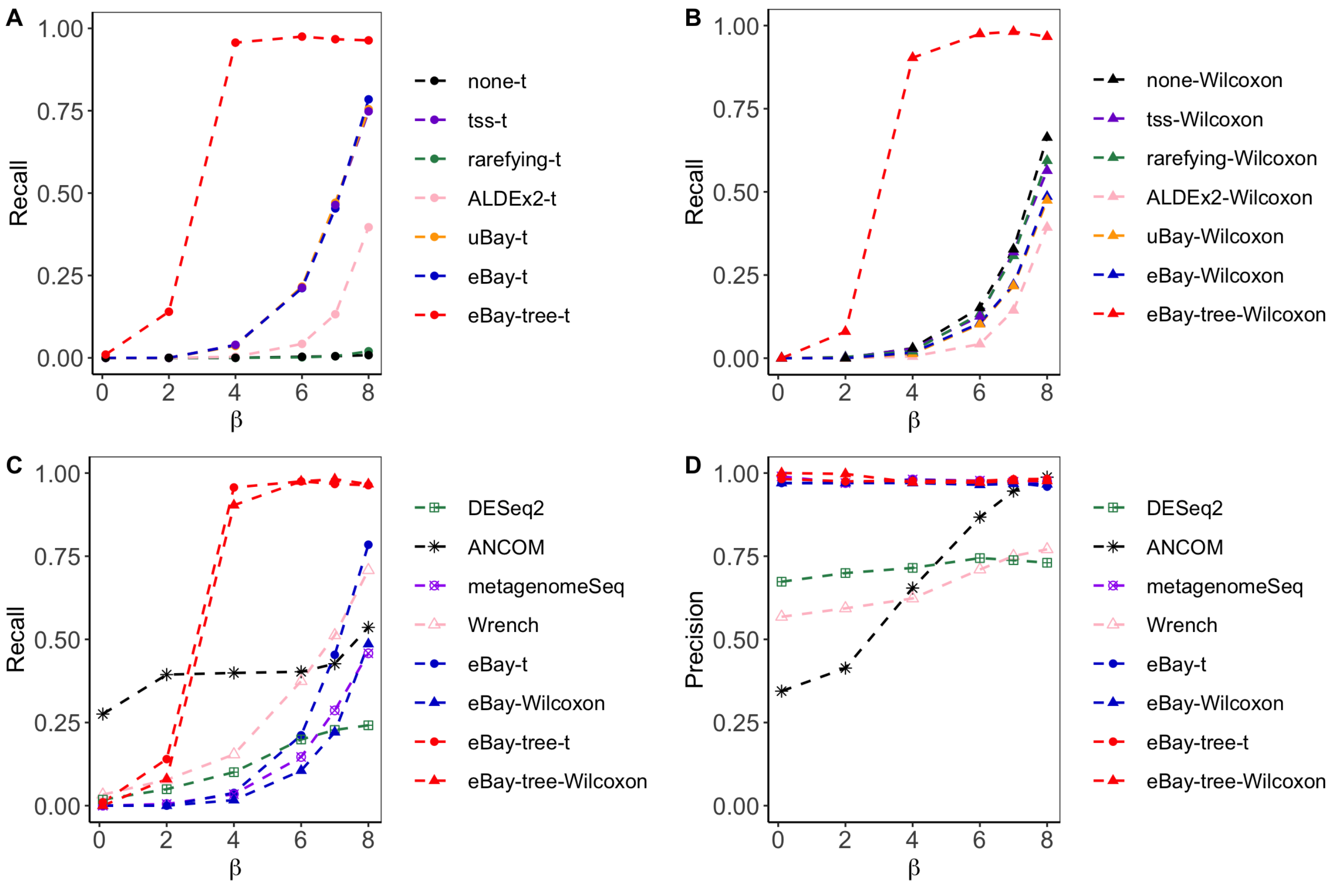

Figure S2 Comparison of recall and precision with data generated from the DTM model across different β: the non-degenerate case. We simulated 100 data sets from the DTM model with $\theta = 0.27$ and $\beta \in \{0.1, 2, 4, 6, 7, 8\}$. **a** and **b** Recall of t-test and Wilcoxon rank sum test with various normalization methods. **c** and **d** Recall and precision of DESeq2, ANCOM, metagenomeSeq, Wrench, and those of t-test and Wilcoxon rank sum test, both applied after counts were normalized by eBay or eBay-tree.

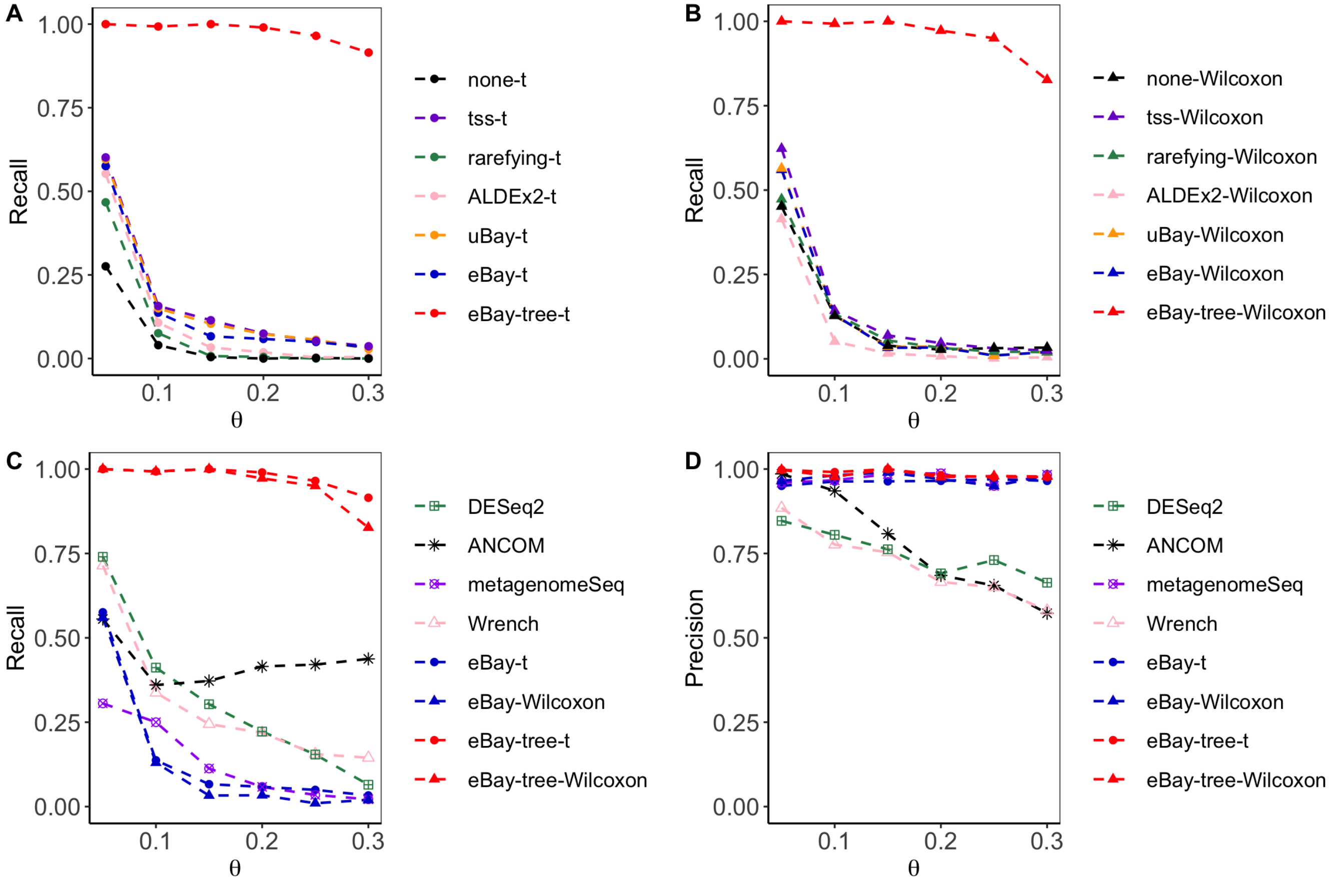

Figure S3 Comparison of recall and precision with data generated from the DTM model across different θ: the non-degenerate case. We simulated 100 data sets from the DTM model with $\beta = 4$ and $\theta \in \{0.05, 0.1, 0.15, 0.2, 0.25, 0.3\}$. **a** and **b** Recall of t-test and Wilcoxon rank sum test with various normalization methods. **c** and **d** Recall and precision of DESeq2, ANCOM, metagenomeSeq, Wrench, and those of t-test and Wilcoxon rank sum test, both applied after counts were normalized by eBay or eBay-tree.

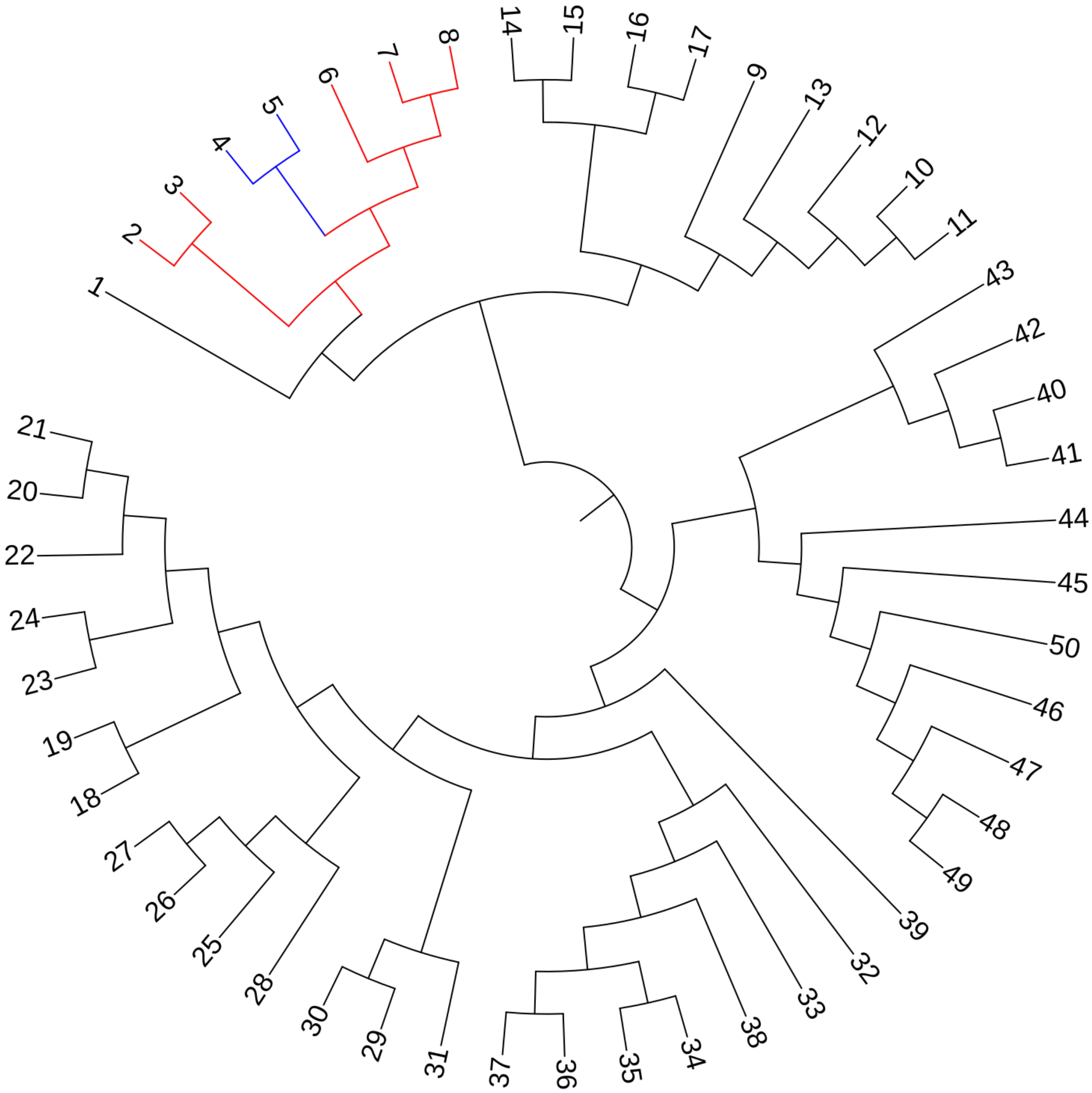

Figure S4 The degenerate case. Two pairs of nodes, $\{55, 56\}$ and $\{57, 58\}$, were set to be differentially abundant, but only 5 leaf nodes, labeled as 2, 3, 6, 7, 8, inherited the differences.

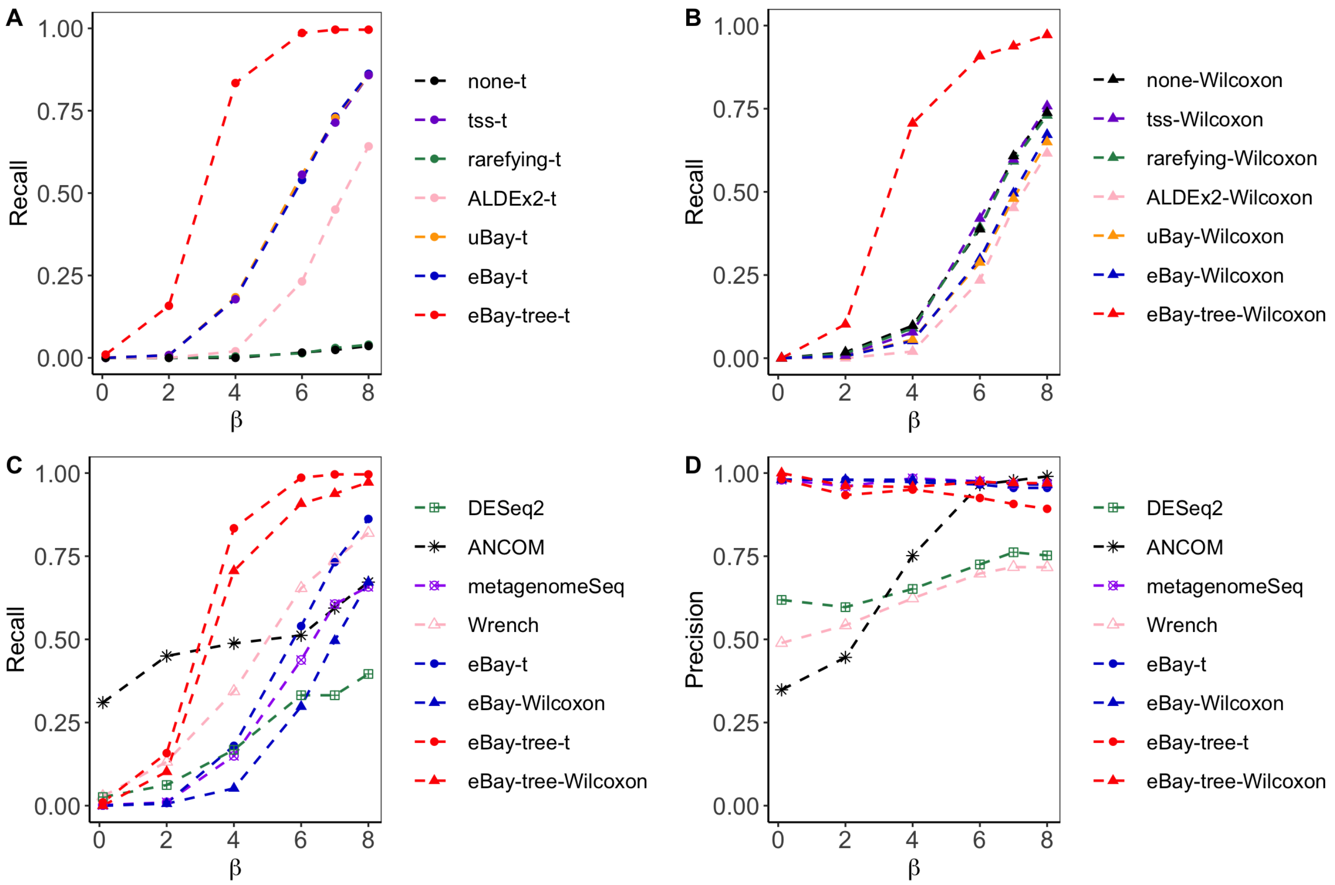

Figure S5 Comparison of recall and precision with data generated from the DTM model across different β: the degenerate case. We simulated 100 data sets from the DTM model with $\theta = 0.27$ and $\beta \in \{0.1, 2, 4, 6, 7, 8\}$. **a** and **b** Recall of t-test and Wilcoxon rank sum test with various normalization methods. **c** and **d** Recall and precision of DESeq2, ANCOM, metagenomeSeq, Wrench, and those of t-test and Wilcoxon rank sum test, both applied after counts were normalized by eBay or eBay-tree.

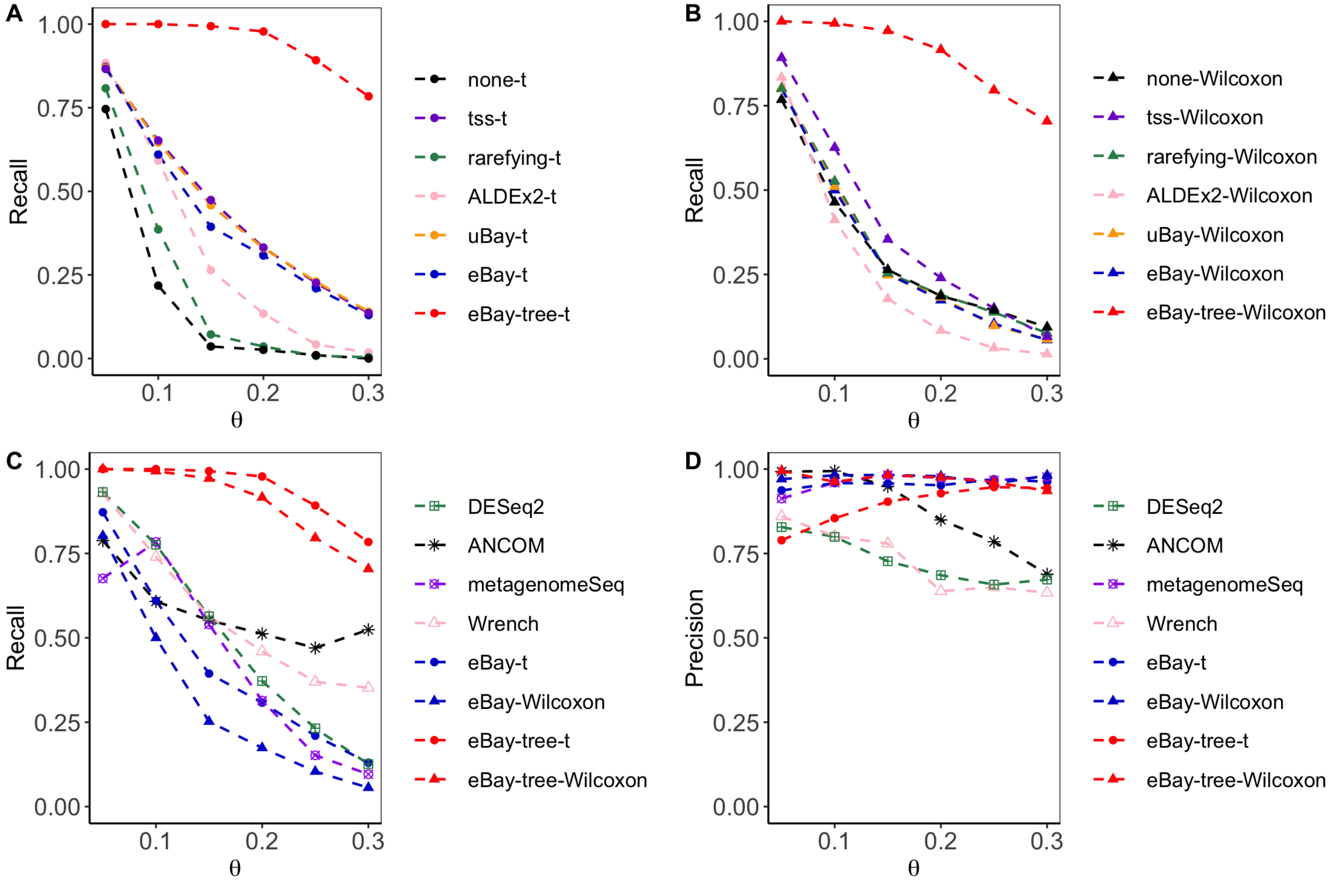

Figure S6 Comparison of recall and precision with data generated from the DTM model across different θ: the degenerate case. We simulated 100 data sets from the DTM model with $\beta = 4$ and $\theta \in \{0.05, 0.1, 0.15, 0.2, 0.25, 0.3\}$. **a** and **b** Recall of t-test and Wilcoxon rank sum test with various normalization methods. **c** and **d** Recall and precision of DESeq2, ANCOM, metagenomeSeq, Wrench, and those of t-test and Wilcoxon rank sum test, both applied after counts were normalized by eBay or eBay-tree.

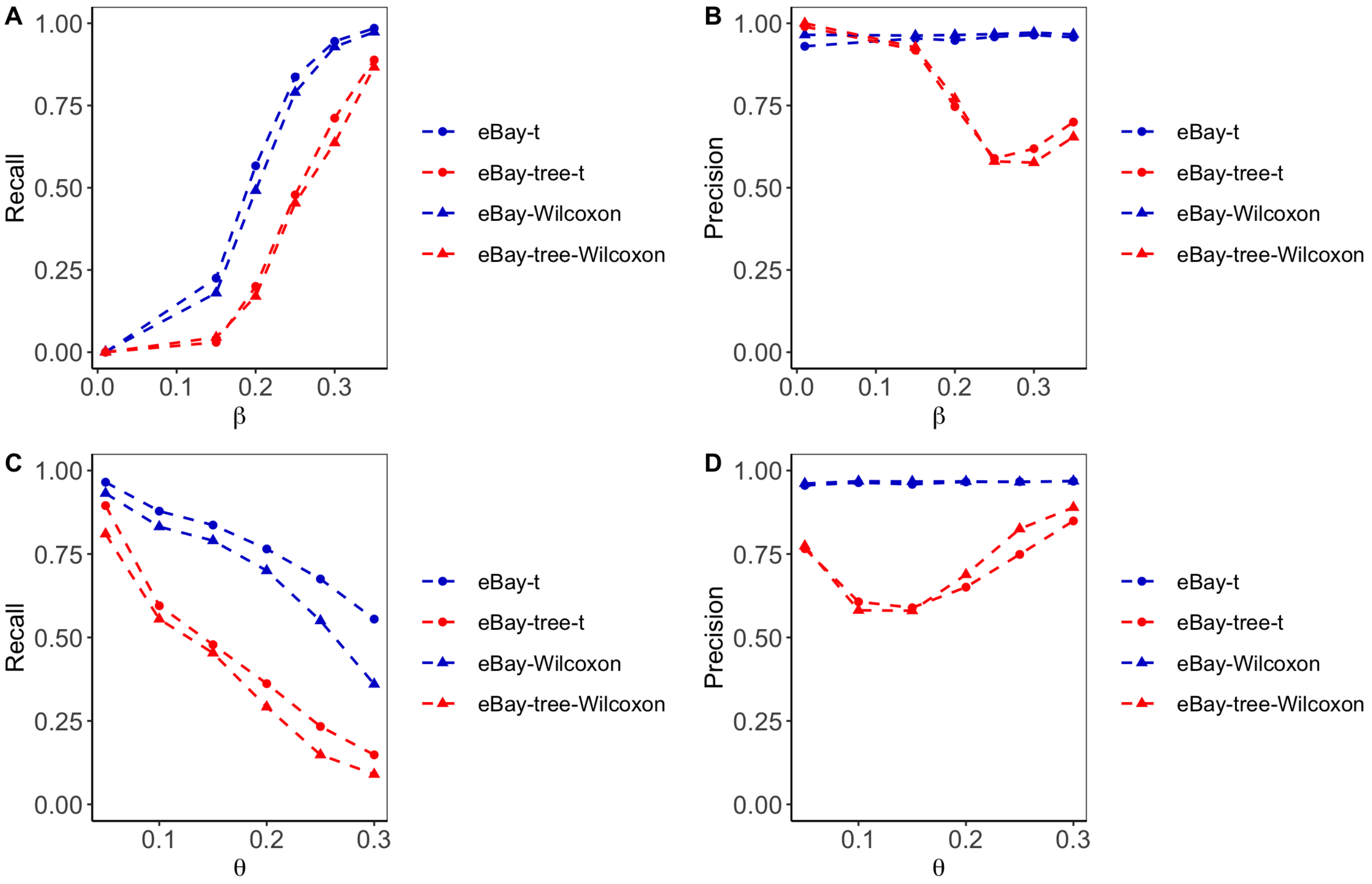

Figure S7 Comparison of recall and precision between eBay and eBay-tree. To detect differentially abundant taxa, we simulated 100 data sets from the DM model. Counts were normalized by eBay or eBay-tree. **a** and **b** $\theta = 0.15$ and $\beta \in \{0.01, 0.15, 0.2, 0.25, 0.3, 0.35\}$. **c** and **d** $\beta = 0.25$ and $\theta \in \{0.05, 0.1, 0.15, 0.2, 0.25, 0.3\}$.

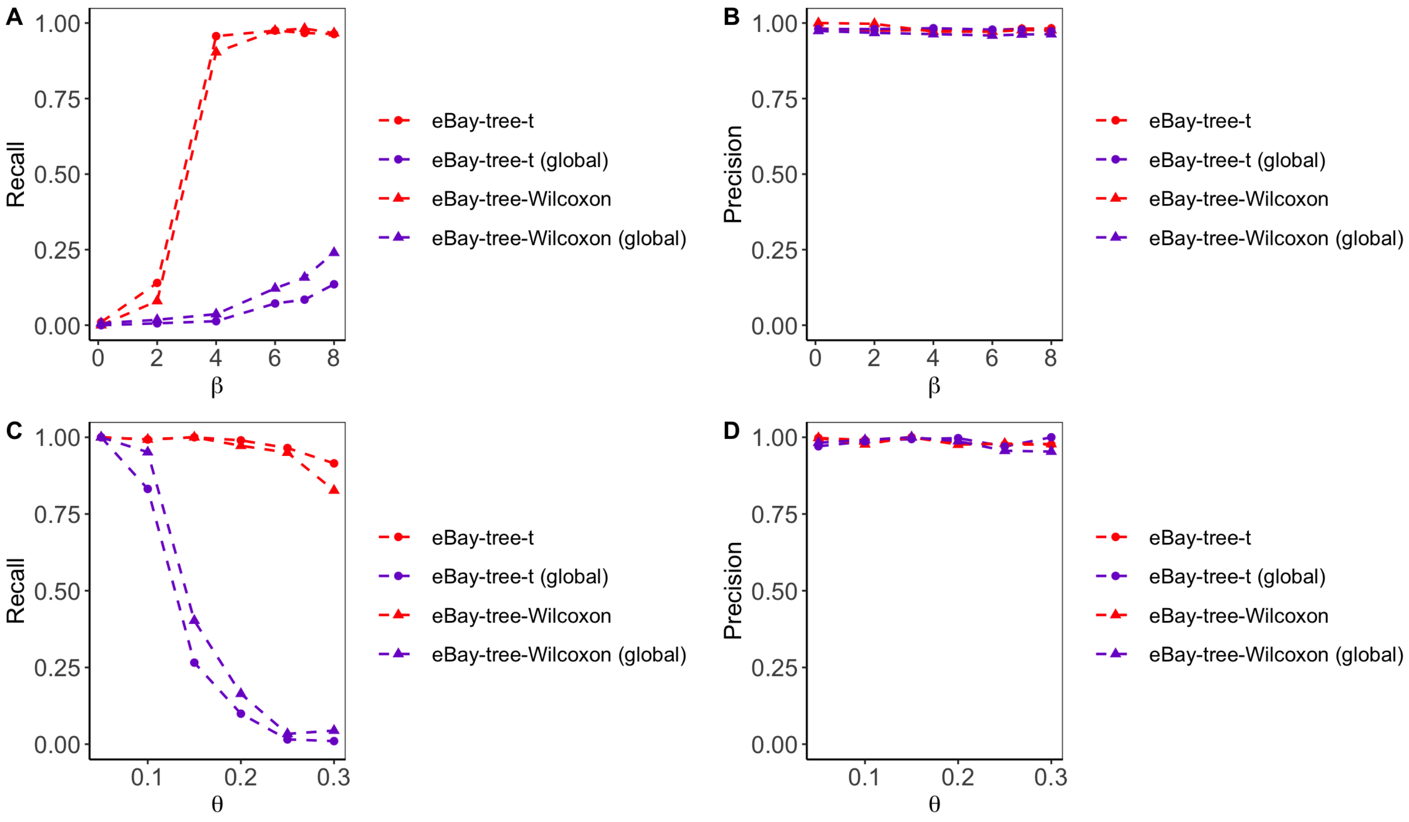

Figure S8 Comparison of recall and precision between eBay-tree and eBay-tree (global). To detect differentially abundant taxa for the non-degenerate case, we simulated 100 data sets from the DTM model. Counts were normalized by eBay-tree. Rather than do the test globally on the normalized data at the leaf-node level (purple), our phylogeny-ware detection procedure carries out local tests at tree splits (red). **a** and **b** $\theta = 0.27$ and $\beta \in \{0.1, 2, 4, 6, 7, 8\}$. **c** and **d** $\beta = 4$ and $\theta \in \{0.05, 0.1, 0.15, 0.2, 0.25, 0.3\}$.

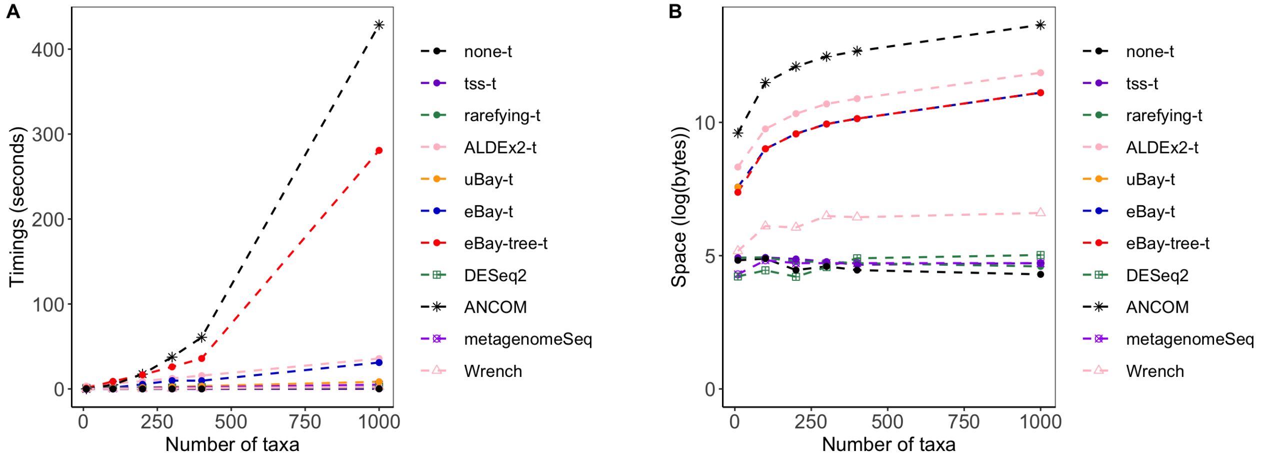

Figure S9 Timings (seconds) and space (log(bytes)), averaged over 10 runs with data generated from the DTM model with $n_1 = n_2 = 50$, versus the number of taxa.

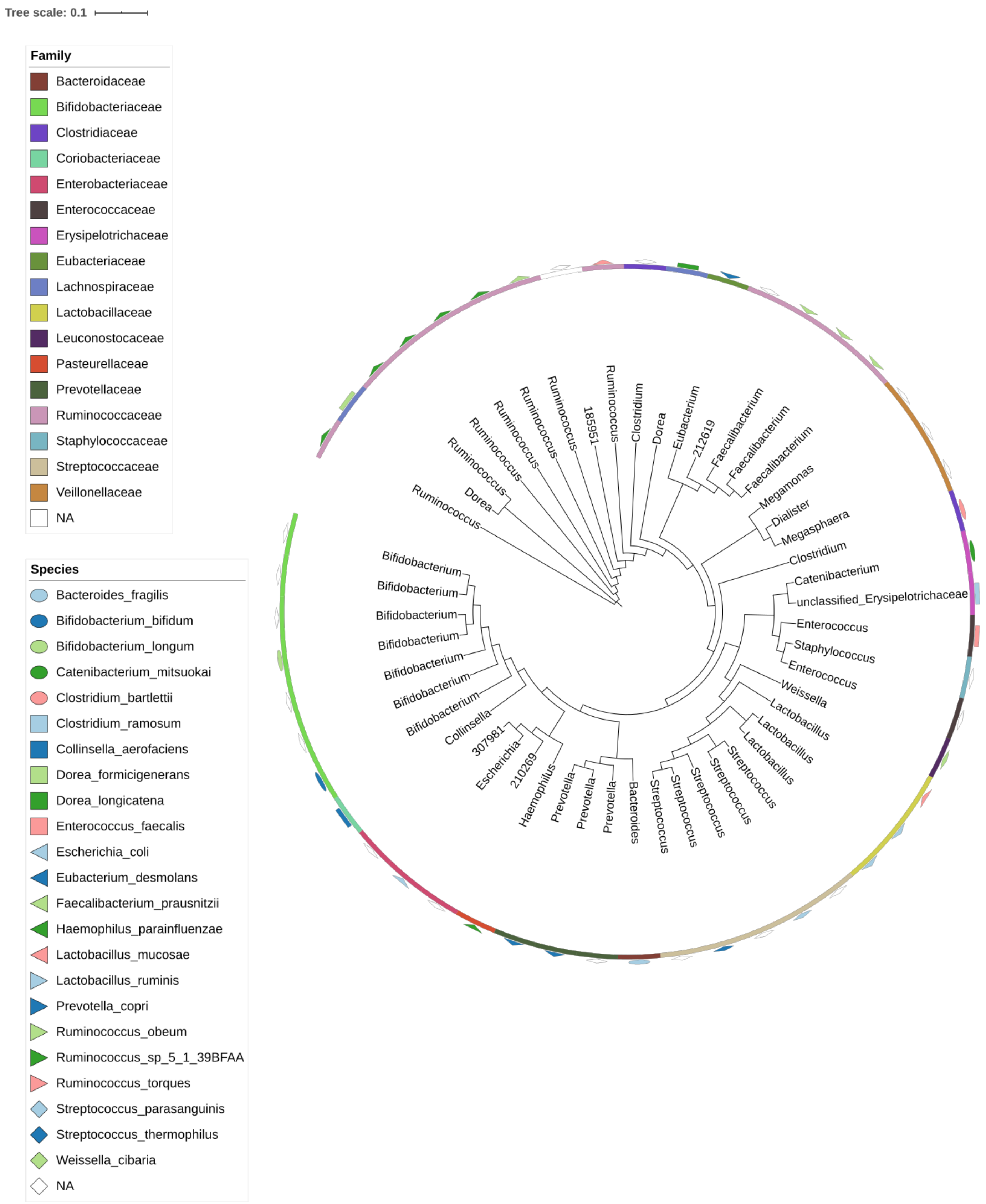

Figure S10 The phylogenetic tree of 50 bacterial taxa inferred by maximum likelihood. We performed sequence alignment and built the tree using PyNAST and FastTree, respectively.

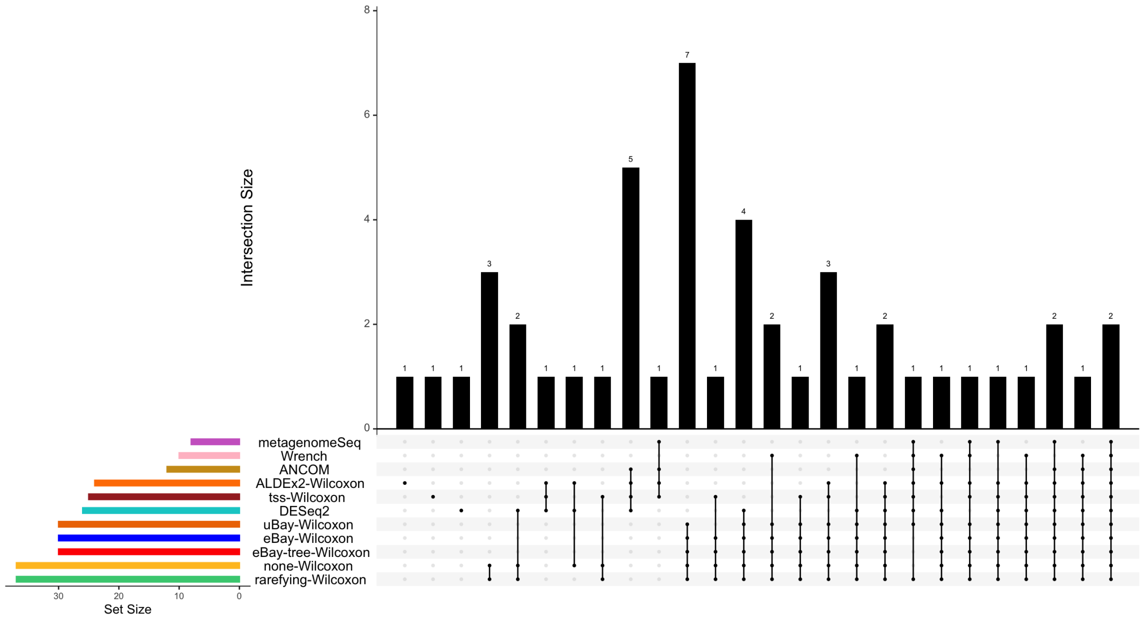

(a)

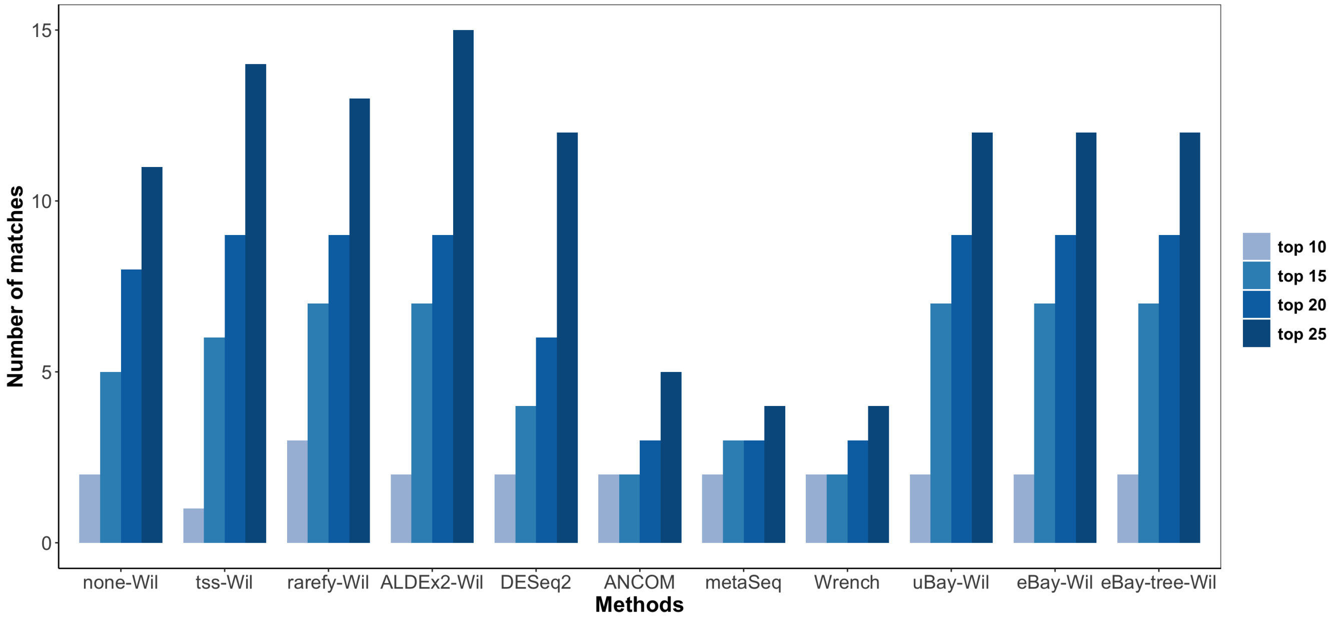

(b)

Figure S11 Differentially abundant species detected by Wilcoxon rank sum test based on the tree in Figure S10. (a) Visualization of set intersections among normalization methods in Table 1 and differential abundance testing methods in Table 2. (b) The number of matches between the top K taxa identified by random forests and the top K differentially abundant taxa detected by various testing methods. Wil: Wilcoxon, metaSeq: metagenomeSeq.

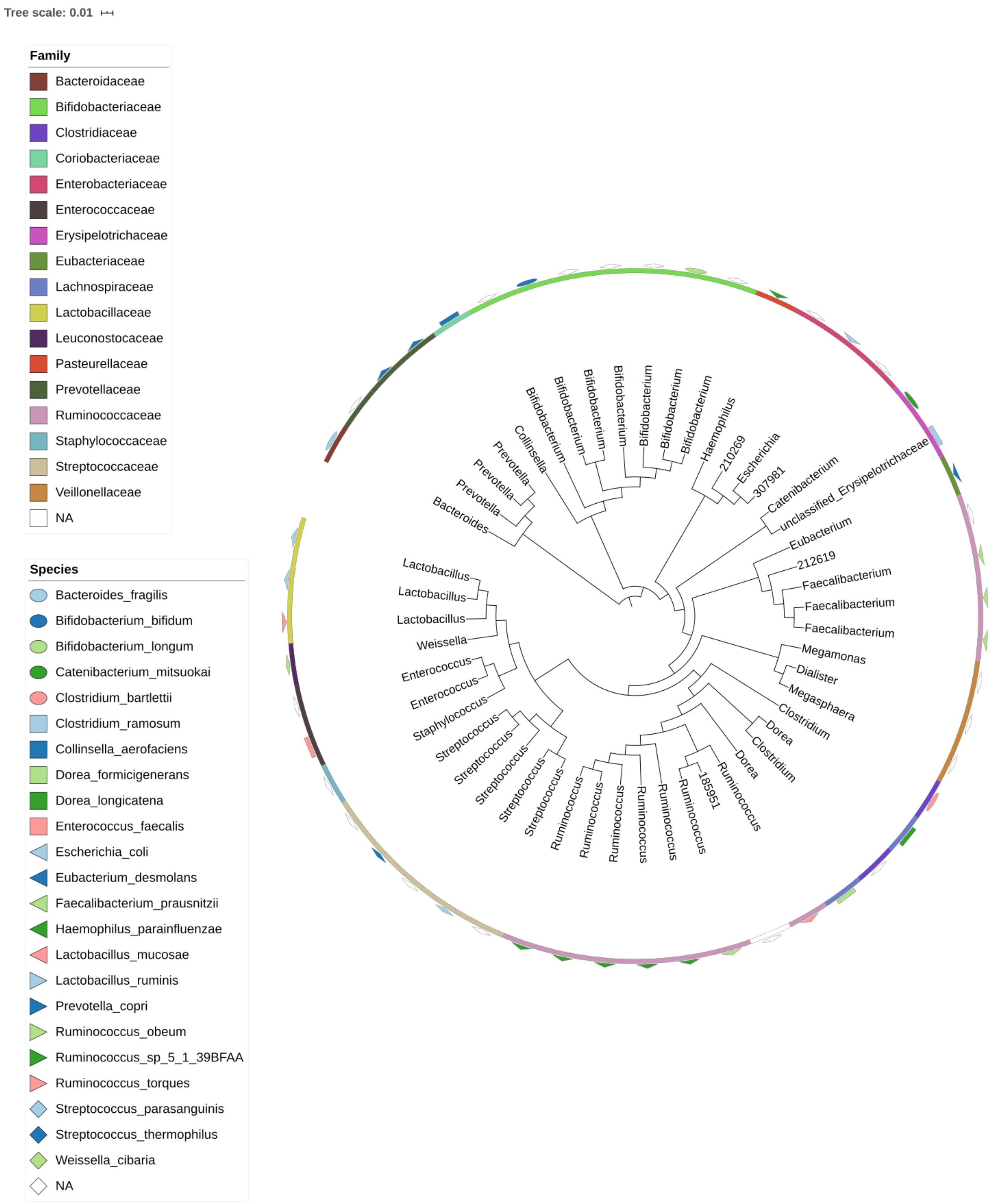

Figure S12 The phylogenetic tree of 50 bacterial taxa built based on distances. We computed the distances between any two species and constructed the tree using the neighbor-joining method in MEGA7.

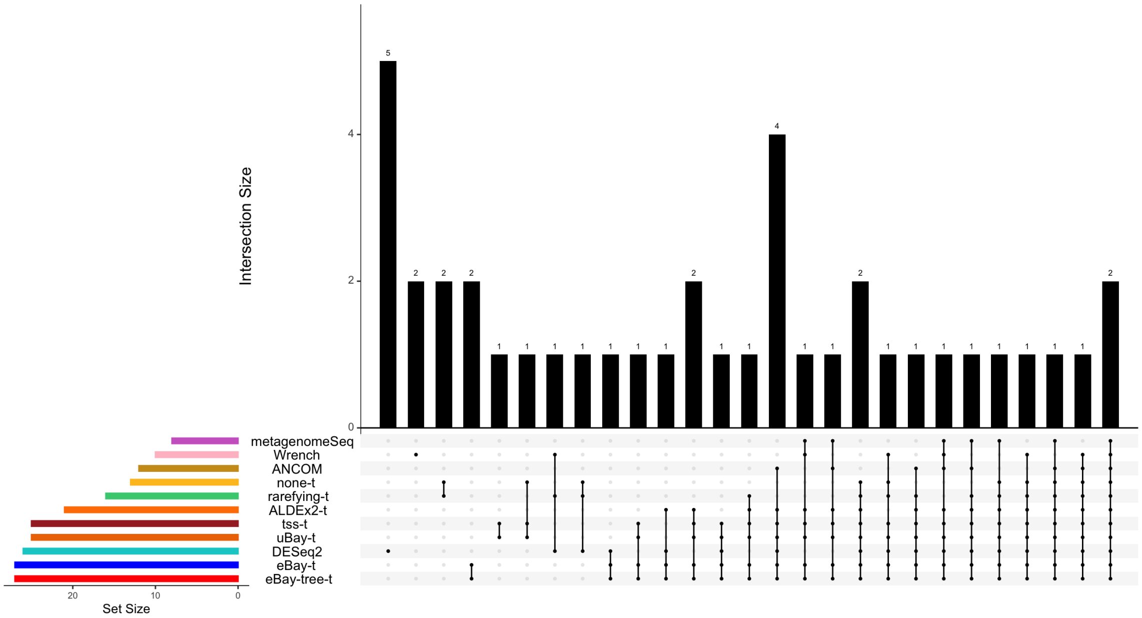

(a)

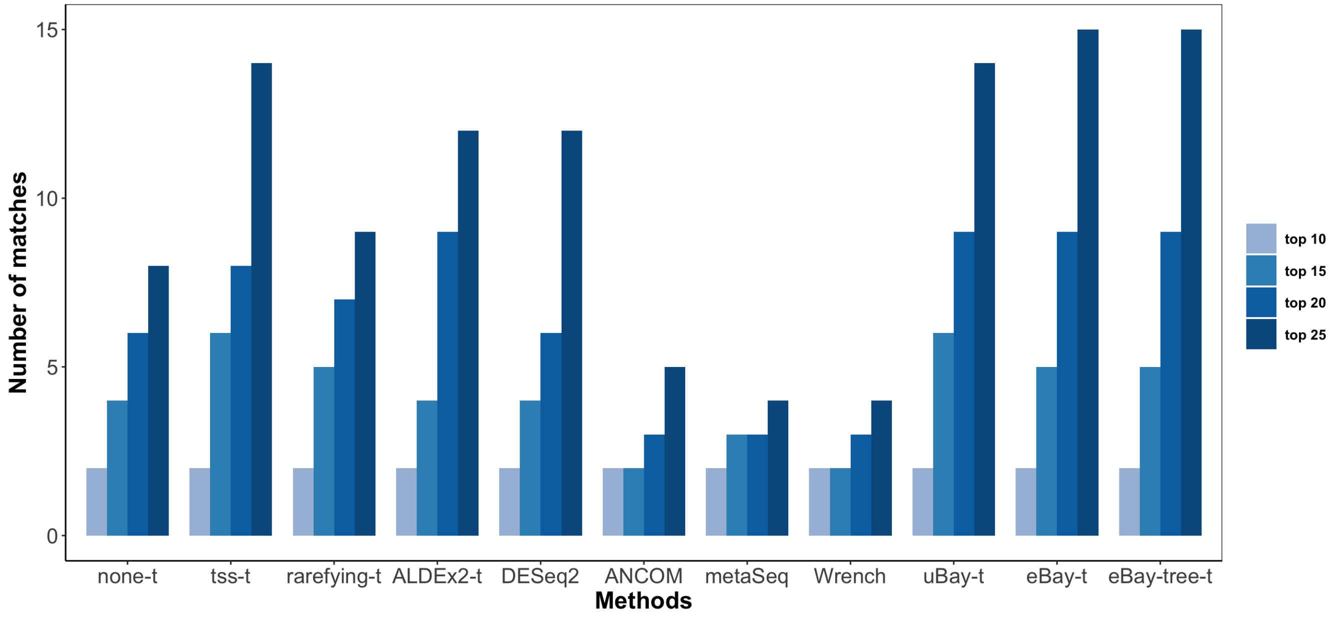

(b)

Figure S13 Differentially abundant species detected by t-test based on the tree in Figure S12. The results were obtained in the same way as in Figure 4, except that the tree in Figure S12 was used for the phylogeny-aware detection procedure. (a) Visualization of set intersections among normalization methods in Table 1 and differential abundance testing methods in Table 2. (b) The number of matches between the top K taxa identified by random forests and the top K differentially abundant taxa detected by various testing methods. metaSeq: metagenomeSeq.

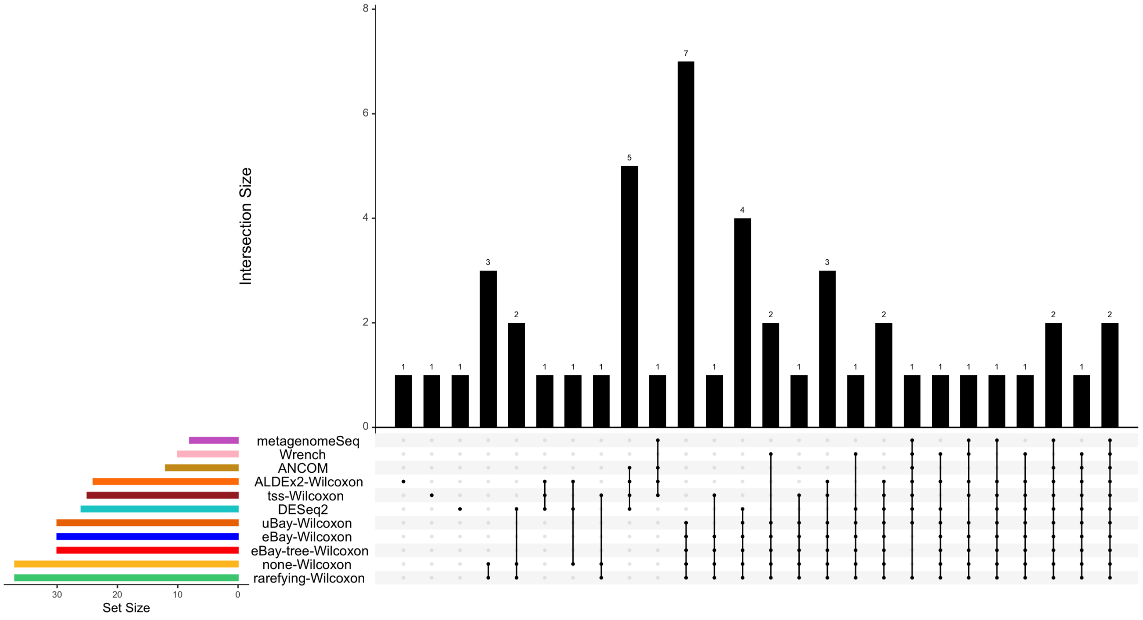

(a)

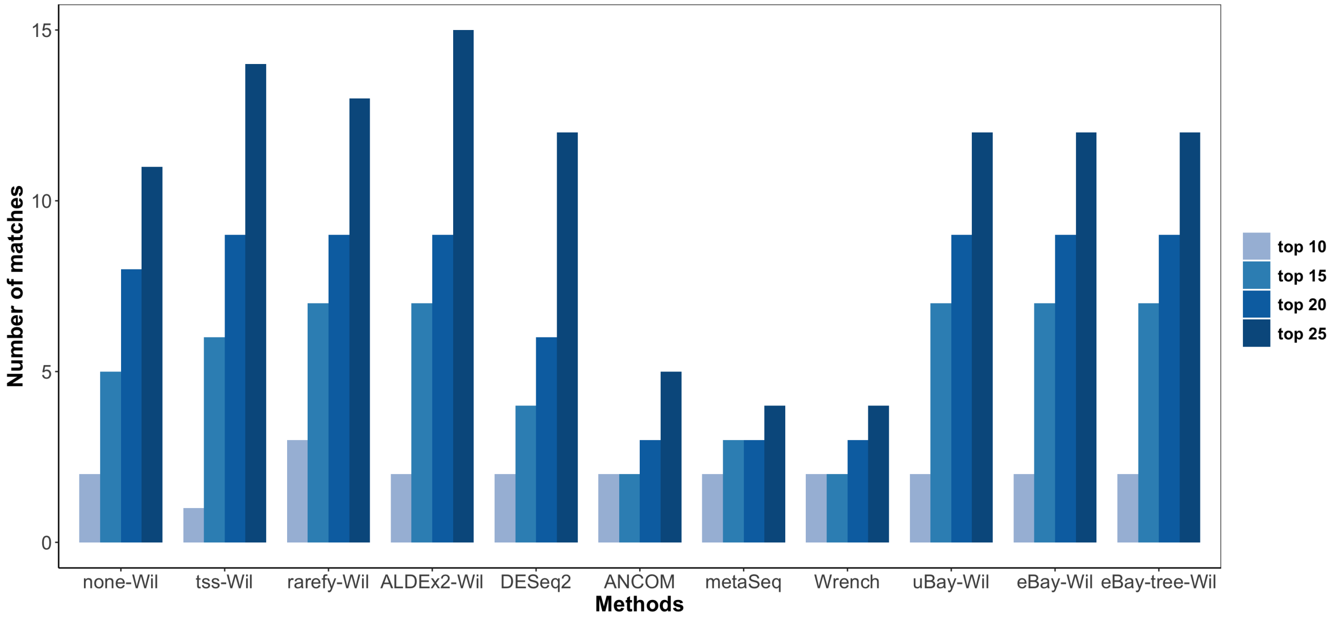

(b)

Figure S14 Differentially abundant species detected by Wilcoxon rank sum test based on the tree in Figure S12. The results were obtained in the same way as in Figure S11, except that the tree in Figure S12 was used for the phylogeny-aware detection procedure. (a) Visualization of set intersections among normalization methods in Table 1 and differential abundance testing methods in Table 2. (b) The number of matches between the top K taxa identified by random forests and the top K differentially abundant taxa detected by various testing methods. Wil: Wilcoxon, metaSeq: metagenomeSeq.
